# Super‐Resolution Ultrasound Based Cell Tracking With Polymeric Nanobubbles

**DOI:** 10.1002/adma.73639

**Published:** 2026-06-06

**Authors:** Junlin Chen, Xiaoyu Wang, Jilin Fan, Bi Wang, Hanghang Fang, Yurui Wang, Hao Cui, Mohammad Roufarshbaf, Ekaterina Savina, Alexandra Valeske, Quim Peña, Yang Shi, Andreas Herrmann, Twan Lammers, Mathias Hornef, Roman Barmin, Thomas Lisson, Georg Schmitz, Anne Rix, Fabian Kiessling

**Affiliations:** ^1^ Institute For Experimental Molecular Imaging RWTH Aachen University Aachen Germany; ^2^ Institute of Technical and Macromolecular Chemistry RWTH Aachen University Aachen Germany; ^3^ DWI – Leibniz‐Institute for Interactive Materials Aachen Germany; ^4^ Institute of Medical Microbiology Rheinisch‐Westfälische Technische Hochschule Aachen University Hospital Aachen Germany; ^5^ Chair for Medical Engineering Ruhr University Bochum Bochum Germany

**Keywords:** cell tracking, intracellular labeling, cell delivery, polymeric nanobubbles, super‐resolution ultrasound, ultrasound localization microscopy

## Abstract

Tracking the fate of transplanted cells in vivo remains a major challenge for the development and monitoring of cell‐based therapies. Here, we report a nanobubble (NB) based acoustic labeling strategy that enables ultrasound localization microscopy (ULM) to track transplanted cells in tumors with single‐cell‐scale sensitivity and super‐resolution spatial precision. We developed stable, biocompatible poly(butyl cyanoacrylate) NB that are efficiently internalized by cells and generate strong intracellular ultrasound signals. Furthermore, ULM allows the real‐time visualization of individual cells, their localization, and trajectory reconstruction in flow phantoms and murine breast cancers. In the latter, NB‐labeled bone marrow‐derived mononuclear cells were tracked in vivo following intra‐arterial injection, whereas unlabeled cells remained undetectable. By integrating cell trajectories with microbubble‐based super‐resolution vascular maps, our approach allows identification of the blood vessels that delivered the cells. This clinically compatible imaging strategy expands the application of ULM beyond vascular mapping and provides a platform for high‐resolution, dynamic monitoring of cell‐based therapies.

## Graphical abstract

This study presents a super‐resolution ultrasound platform for tracking cells in vivo. Biocompatible polymeric nanobubbles are used as highly echogenic intracellular labels. Following the injection of cells and microbubbles, ultrasound localization microscopy (ULM) can dynamically match the microvascular architecture and individual cell trajectories. This non‐invasive approach allows for the precise, high‐resolution monitoring of therapeutic cell delivery within the tumour microenvironment.

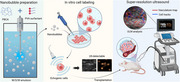

## 1 Introduction

Cell‐based therapies show great promise for various diseases, in particular for tissue regeneration or immune modulation [1, 2, 3, 4]. However, translating these therapies into clinical practice remains challenging. One of the key bottlenecks is the difficulty in tracking where the administered cells go and whether they reach their intended targets [5]. Reliable cell tracking tools are therefore important, not only for understanding therapeutic mechanisms but also for optimizing dosing, administration routes, and safety assessments.

Several medical imaging techniques have been explored to address this need. Among them, magnetic resonance imaging (MRI) offers excellent soft‐tissue contrast and high spatial resolution. However, its sensitivity and specificity for detecting small populations of labeled cells remain limited (>10^3^–10^5^ cells/voxel) [6]. Moreover, cell labeling for MRI commonly relies on iron oxide or gadolinium‐based contrast agents, each presenting inherent drawbacks: iron oxide nanoparticles produce negative contrast that hinders precise cell identification (i.e., differentiation from susceptibility artifacts is challenging) and quantification, while gadolinium‐based agents suffer from poor biocompatibility and potential cytotoxicity [7, 8]. Positron emission tomography (PET) and single‐photon emission computed tomography (SPECT) can provide higher sensitivity (usually detecting >10^2^ cells per voxel, and enabling single‐cell‐level detection under optimized preclinical conditions) and allow quantitative evaluation, yet their dependence on radioactive tracers raises concerns regarding repeated monitoring and long‐term safety [9, 10, 11, 12]. As an alternative, bioluminescence imaging (BLI) is well suited for assessing viable cell assemblies in small‐animal studies, but it requires genetic modification and suffers from poor tissue penetration, making it impractical for human use [13, 14]. Taken together, these methods each provide valuable information but struggle to meet the combined requirements of biocompatibility, real‐time imaging, sensitivity, and clinical translatability that cell‐based therapy demands.

Ultrasound (US) imaging offers an attractive alternative, which is cost‐effective and capable of real‐time imaging without ionizing radiation. Furthermore, it can be easily integrated into clinical workflows [15]. Despite these advantages, the US has not been extensively explored for cell‐tracking applications, primarily due to several key challenges. First, transplanted cells generally exhibit acoustic impedances similar to those of surrounding tissues, making it difficult to detect them by US [16]. Recent studies have demonstrated that engineering therapeutic cells with gas‐filled bubbles, vaporizing phase‐change nanodroplets, or employing genetic acoustic reporter systems can markedly enhance their US contrast [17, 18, 19]. However, nanodroplet‐based approaches often rely on destructive vaporization pulses that perturb tissue and prevent longitudinal monitoring, while genetic acoustic reporters face limitations in manufacturability, expression efficiency in mammalian cells, and stringent regulatory hurdles [5]. Consequently, these strategies are not yet suitable for clinical cell‐tracking applications. In contrast, gas‐filled bubbles have gained growing interest and are currently under extensive preclinical evaluation [16, 20]. Nevertheless, no viable formulation for cell labeling has yet been developed, as existing bubble shells exhibit poor stability and are prone to coalescence under ultrasound exposure [21, 22].

Moreover, the advent of ultrasound localization microscopy (ULM) has revolutionized vascular ultrasound imaging. ULM is a super‐resolution technique that localizes and tracks individual microbubbles over time to map microvascular structures with micrometer precision, surpassing the diffraction limit and achieving substantially higher spatial resolution than conventional diagnostic US [23, 24]. This capability also opens new possibilities for detecting and tracking labeled single cells in vivo. However, its applicability to cell tracking has not yet been demonstrated.

In this study, we fabricated stable polymeric nanobubbles (NB) as a cell labeling agent and explored their potential for cell tracking in combination with ULM. Furthermore, we investigated whether the combination of ULM vascular maps and cell trajectories enables the identification of the vessels that deliver the cells into tumors. We hypothesized that, when combined with ULM, this approach could enable single‐cell‐level detection, providing exceptional sensitivity and spatial precision for monitoring therapeutic cells in vivo and paving the way toward real‐time, quantitative, and clinically feasible cell tracking.

## 2 Results

### 2.1 Preparation and Characterization of PBCA NB

**SCHEME 1:**
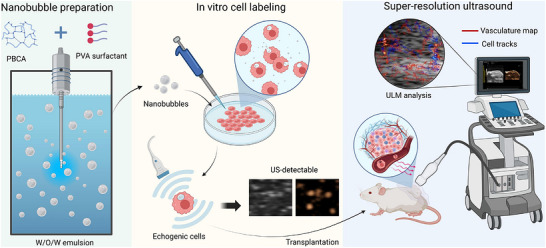
Schematic overview of cell tracking using NB and ULM. Biocompatible poly(butyl cyanoacrylate) (PBCA) NB was generated using a double emulsion formulation and subsequently applied for in vitro labeling of transplanted cells. NB‐labeled cells were detectable by both B‐mode and nonlinear contrast mode US. Following intravascular cell injection into tumor‐bearing mice, dynamic US imaging enabled the visualization of cell migration into the tumor microenvironment. ULM post‐processing allowed the reconstruction of super‐resolution maps: microbubble‐based vascular architecture and individual NB‐labeled cell trajectories. This combined acoustic labeling and ULM strategy enables high‐resolution tracking of the delivery of cells on super‐resolution vascular maps. The schematic was created with BioRender.com.

**FIGURE 1:**
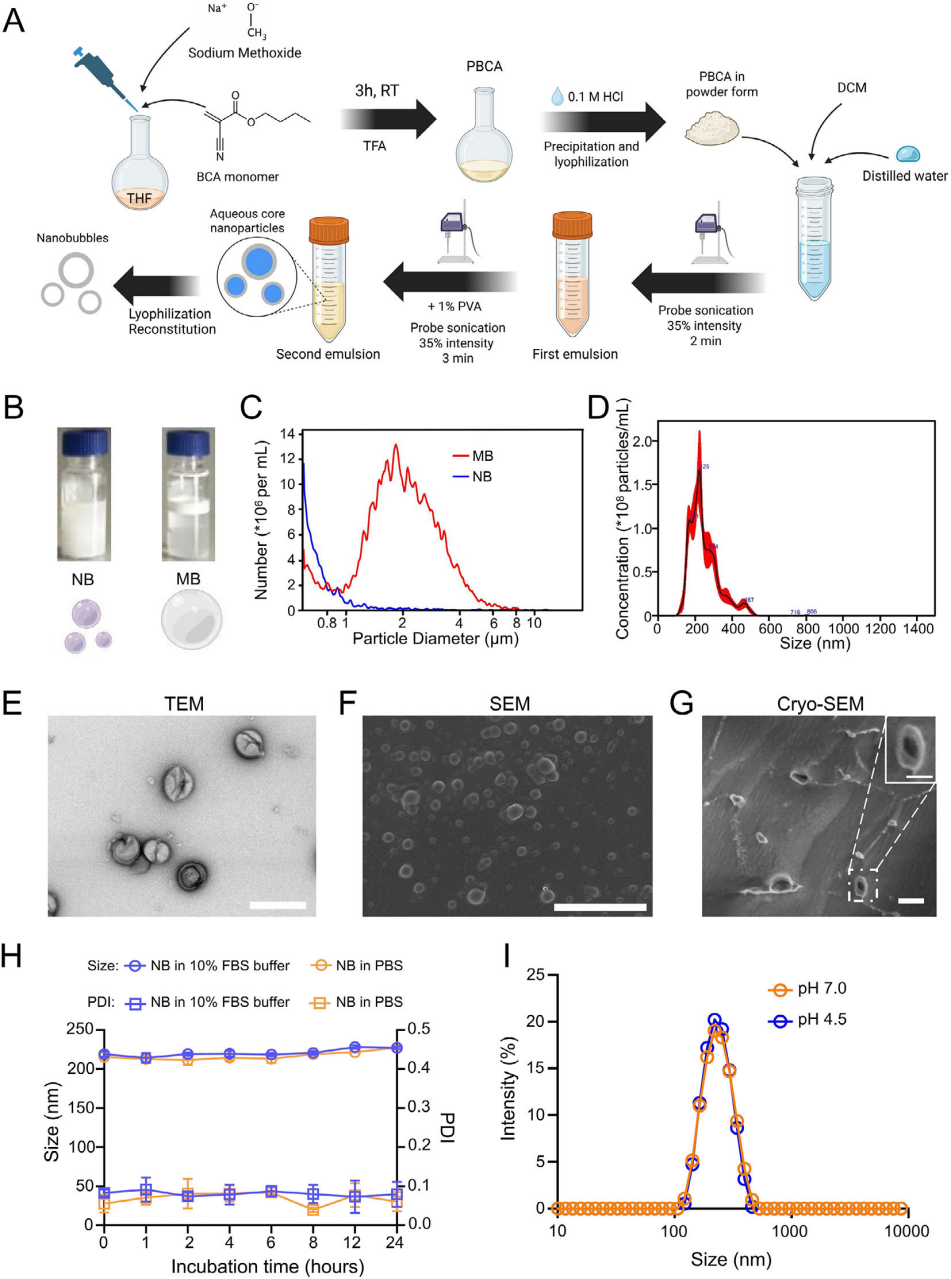
Fabrication, physicochemical characterization, and stability of PBCA NB. (A) The Schematic illustration depicts the fabrication of PBCA NB. The schematic was created with BioRender.com. (B) Representative photographs of PBCA NB and MB suspensions after standing for 24 h show that MB forms a floating cake due to buoyancy, whereas NB remains homogeneously dispersed. (C) Coulter counter measurements of MB (red) and NB (blue) samples show a dominant micrometer‐scale size distribution for MB centered in the 1–4 µm range, whereas no detectable microscale population is observed for NB. (D) Nanoparticle tracking analysis (NTA) shows the size distribution and concentration of PBCA NB in the nanoscale range. (E) A representative transmission electron microscopy image (TEM, scale bar: 500 nm) shows predominantly spherical, bubble‐like PBCA NB, with partial collapse attributed to sample preparation conditions. (F) A representative scanning electron microscopy image (SEM, scale bar: 2 µm) shows spherical, bubble‐like PBCA NB. (G) A representative Cryo‐SEM image of fractured PBCA NB (scale bar: 500 nm; zoom‐in scale bar: 300 nm) reveals distinct internal cavities. (H) DLS measurements of hydrodynamic diameter and PDI over 24 h at 37°C show that PBCA NB remain stable in PBS and in PBS containing 10% FBS (*n = 3*). Data are shown as mean ± SD. Statistical significance was determined by one‐way ANOVA with Tukey's post hoc test. No statistically significant differences were observed across the evaluated conditions (*p* > 0.05). (I) DLS size distributions of the NB measured after 24 h of incubation at pHs 7.0 and 4.5 show no significant changes, indicating stability under acidic conditions relevant to intracellular (lysosomal) environments.

To engineer echogenic cells, we leveraged nanoscale gas‐filled bubbles as intracellular acoustic labels. Owing to their nanoscale dimensions, NB can be efficiently internalized by cells, enabling subsequent in vivo cell‐tracking applications (Scheme 1). In this study, we focused on a poly(butyl cyanoacrylate) (PBCA) based formulation optimized for US imaging. PBCA NB were generated using a double‐emulsion approach to form polymeric core–shell nanoparticles, followed by lyophilization to create a hollow, gas‐filled architecture (Figure 1A). For comparison, PBCA microbubbles (MB) were prepared using established protocols [25]. Distinct buoyancy behaviors were observed between the two formulations. After standing for 24 h, MB suspensions exhibited a visible floating cake at the top of the vial due to buoyancy, whereas NB suspensions remained uniformly milky, indicating low buoyancy and homogeneous dispersion in solution (Figure 1B). To exclude microscale bubbles in the NB formulation, Coulter counter analysis was performed. No detectable microscale population was observed in the NB sample (Figure 1C, blue curve). In contrast, the MB sample displayed a pronounced micrometer‐scale population centered in the 1–4 µm range (Figure 1C, red curve). Nanoparticle tracking analysis (NTA) revealed that PBCA NB had a mean diameter of 217 ± 40 nm with a narrow size distribution (PDI = 0.08 ± 0.03) (Figure 1D). During NTA recordings, NB appeared as highly scattering, mobile objects exhibiting dynamic intensity fluctuations and diffuse halos surrounding a bright central signal, suggesting strong refractive index mismatch at the gas–liquid interface (Movie S1). Morphological characterization by transmission electron microscopy (TEM) and scanning electron microscopy (SEM) further showed predominantly spherical, bubble‐like structures (Figure 1E,F). Partial collapse observed in TEM images was attributed to free‐drying and loss of internal gas pressure under vacuum. In contrast, cryogenic SEM preserved the native hydrated state and revealed distinct internal cavities within fractured NB (Figure 1G), providing direct structural evidence of a hollow architecture consistent with NTA observations. Next, the colloidal stability of PBCA NB was evaluated under physiologically relevant conditions using dynamic light scattering (DLS). PBCA NB maintained stable hydrodynamic diameters and low polydispersity indices for up to 24 h when incubated in both phosphate‐buffered saline (PBS) and PBS containing 10% fetal bovine serum (FBS) at 37°C (Figure 1H), indicating resistance to aggregation and coalescence. Moreover, PBCA NB remained stable under acidic conditions (pH 4.5), with comparable distributions observed after 24 h incubation at neutral pH (Figure 1I), supporting their suitability for intracellular labeling.

### 2.2 Efficient Cell Labeling With PBCA NB

**FIGURE 2:**
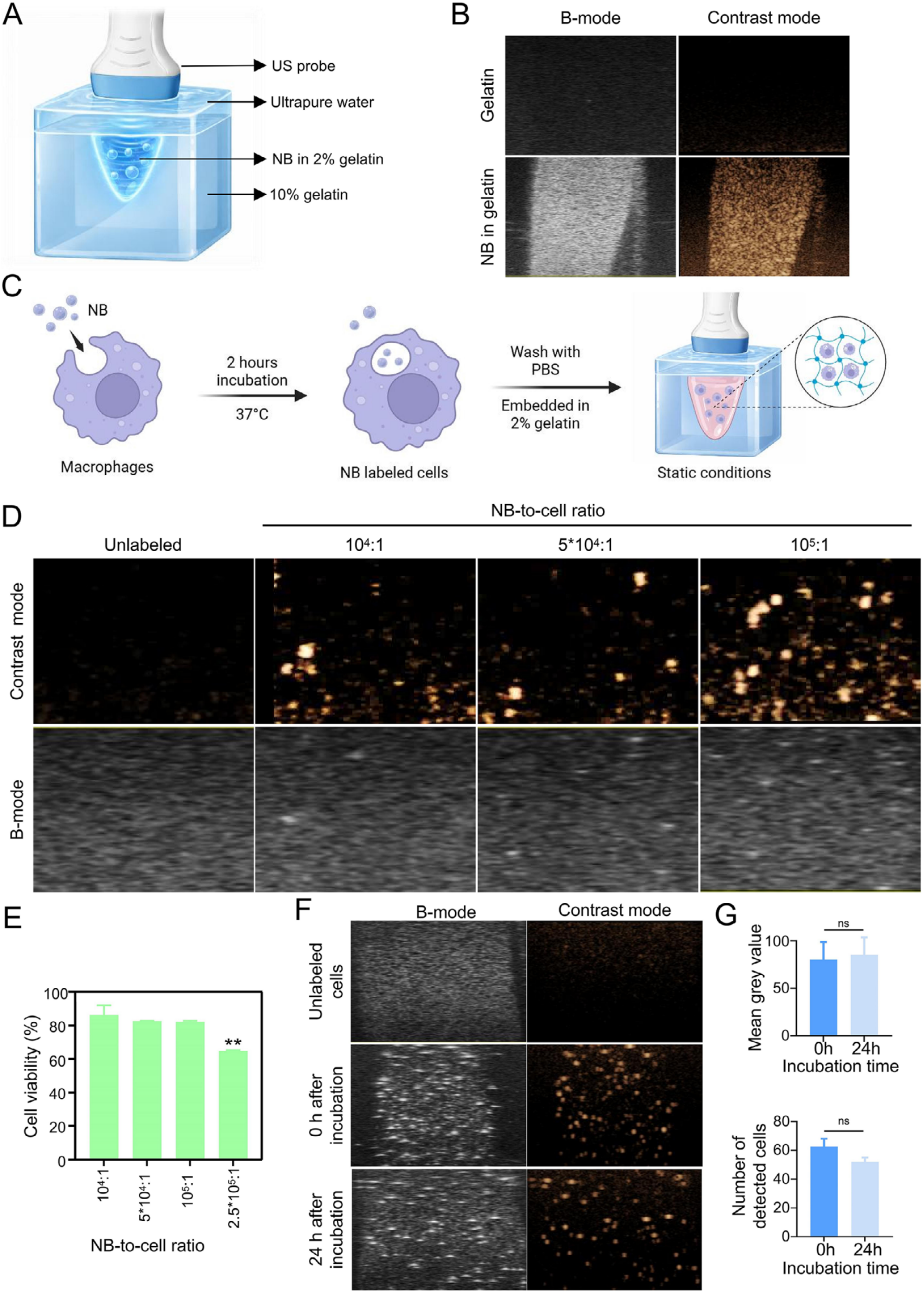
In vitro echogenicity and optimization of cell‐labeling with PBCA NB. (A) This schematic illustrates our gelatin phantom used for US imaging, in which a 10% gelatin matrix serves as a US‐transparent base, and NB dispersed in 2% gelatin is introduced into a preformed hollow well and solidified by cooling, with degassed water used as the coupling medium. (B) Representative B‐mode and contrast‐mode US images of gelatin alone and NB‐containing gelatin demonstrate detectable acoustic signals from PBCA NB. (C) Schematic illustrating the in vitro NB labeling of J774A.1 macrophages and their embedding in a 2% gelatin phantom for static US imaging. (D) Representative B‐mode and contrast‐mode US images show cells labeled with NB at different NB‐to‐cell ratios, with cells labeled at a ratio of 10^5^:1 exhibiting the strongest signal in both imaging modes. (E) Cell viability assessed by XTT assay after NB incubation at varying NB‐to‐cell ratios shows no significant loss of viability at ratios of 10^5^:1 and below (*n = 3*). Data are shown as mean ± SD. Statistical significance was determined using a one‐way ANOVA followed by Tukey's post hoc test (^**^p < 0.01 compared to the 10^4^:1 ratio). (F) Representative US images acquired immediately after NB labeling (0 h) and after 24 h of culture show retained detectability of NB‐labeled cells. (G) Quantification of the detected cell number per imaging plane and mean grey value at 0 and 24 h after labeling shows no statistically significant differences (*n = 3*). Data are shown as mean ± SD. Statistical analysis was performed using an unpaired two‐tailed Student's *t*‐test (*ns* = not significant, *p* > 0.05).

To assess the intrinsic echogenicity of PBCA NB, NB were embedded in a 2% gelatin phantom and imaged by US (Figure 2A). As shown in Figure 2B, NB generated detectable signals in both B‐mode and nonlinear contrast mode, confirming their acoustic visibility under static conditions. Continuous monitoring of NB in a gelatin phantom over an 8 s acquisition period revealed a stable acoustic intensity in contrast mode, confirming that our standard imaging and imaging sequence at 4% intensity is non‐destructive (Figure S1). To further investigate this nonlinear behavior, acoustic scattering measurements in flow conditions using an amplitude modulation sequence were performed, confirming that the NB actively generates nonlinear acoustic echoes under 18 MHz US excitation (Figure S2). We next performed an in vitro comparison of their echogenicity against PBCA MB. Because US scattering is driven by the compressible gas core, we normalized the comparison by matching the total estimated gas volume of the two populations. NB generated approximately ∼5% of the acoustic intensity in contrast mode compared to an equivalent gas volume of MB (Figure S3). Taking benefit from the high phagocytic activity of J774A.1 macrophages [26, 27], we next evaluated the feasibility of intracellular NB labeling in vitro (Figure 2C). Cells were incubated with NB at four different NB‐to‐cell ratios, and viability was quantified using an XTT assay. NB‐to‐cell ratios of 10^4^:1, 5 × 10^4^:1, and 10^5^:1 were well tolerated, yielding viabilities of 86.5 ± 5.5%, 82.7 ± 0.2%, and 82.3 ± 0.6%, respectively (Figure 2E). Increasing the ratio to 2.5 × 10^5^:1 resulted in a significant reduction in viability to 64.0 ± 0.4%, indicating that higher NB loading compromises cell viability. We next examined the echogenicity of NB‐labeled cells embedded in gelatin phantoms at NB‐to‐cell ratios of 10^4^:1, 5 × 10^4^:1, and 10^5^:1. As shown in Figure 2D, cells labeled at a ratio of 10^5^:1 exhibited the strongest signal in contrast mode and B‐mode images. On the basis of these results, an NB‐to‐cell ratio of 10^5^:1 was selected as the optimal labeling condition, providing enhanced acoustic contrast without substantial impact on cell viability. Furthermore, exposing NB‐labeled macrophages to a destructive US pulse did not result in a significant decrease in cell viability, indicating that cellular integrity is preserved even if NB destruction occurs (Figure S4). Finally, we assessed the persistence of NB‐derived US signals in labeled cells over time. Following NB incubation, cells were cultured for an additional 24 h and imaged under identical conditions. NB‐labeled cells remained detectable by both B‐mode and contrast‐mode ultrasound after 24 h of culture (Figure 2F). Quantitative analysis revealed no statistically significant differences in either the number of detected cells per imaging plane (62 ± 5.5 at 0 h vs 52 ± 3.0 at 24 h) or the mean grey value per detected cell (80.2 ± 18.6 vs 85.1 ± 18.5) (Figure 2G). These results indicate that NB‐labeled cells are detectable for more than 24 h. To further highlight the translational potential of our approach, we evaluated the echogenicity of NB‐labeled cells using a clinical US system (Canon Aplio i800, 4 MHz). As acoustic scattering generally increases with frequency, the NB‐labeled cells exhibited lower echogenicity in fundamental B‐mode at 4 MHz compared to the 18 MHz preclinical setup. However, they generated robust and clearly detectable signals in contrast mode (Figure S5). This indicates that NB‐labeled cells provide sufficient non‐linear acoustic contrast outside the preclinical high‐frequency regime.

**FIGURE 3:**
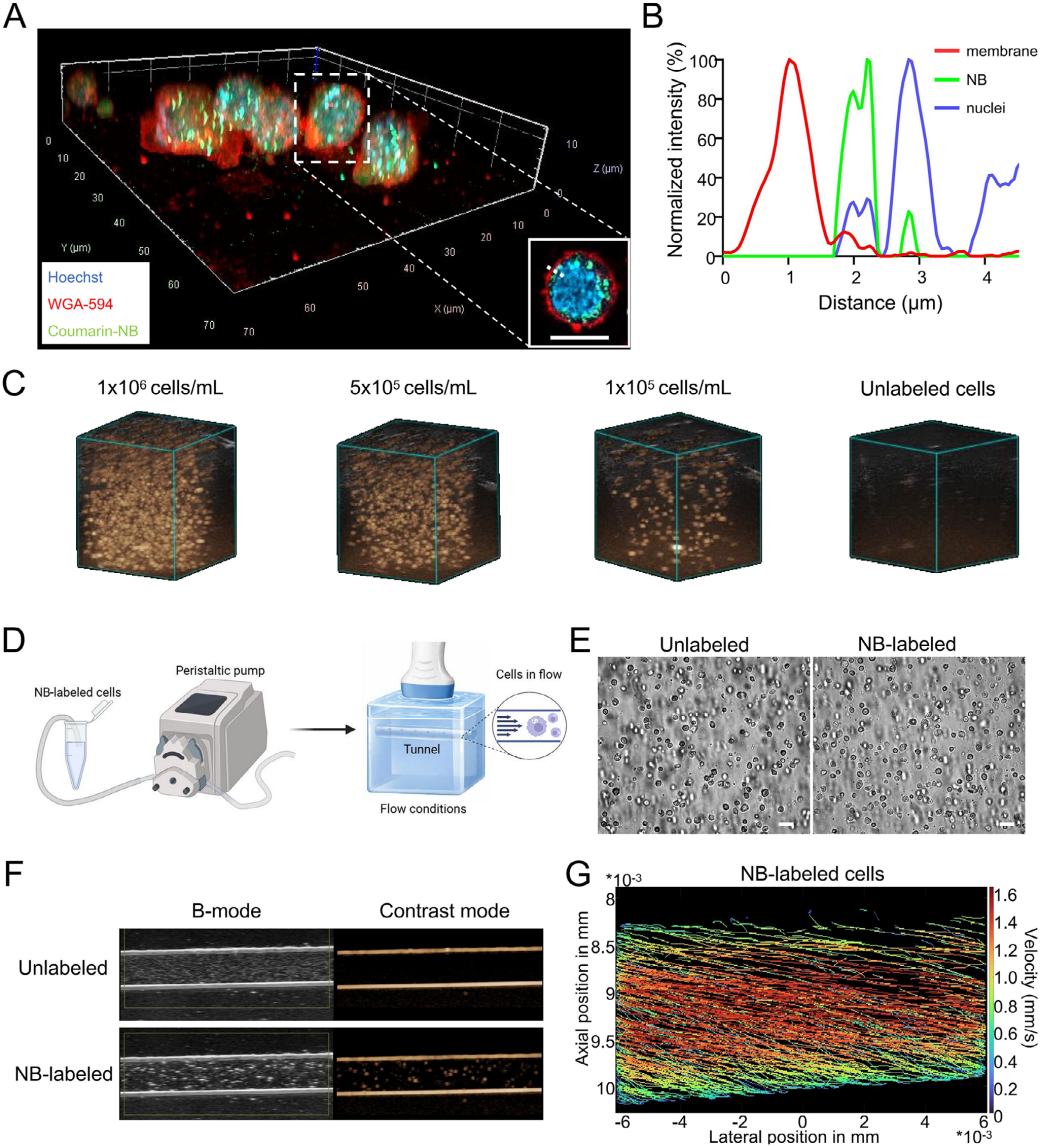
Intracellular localization of NB in cells by confocal microscopy and in vitro cell tracking using ULM. (A) A 3D confocal reconstruction shows intracellular localization of NB‐labeled cells, with NB shown in green, cell membranes in red, and nuclei in blue. NB‐labeled cells show distinct NB‐associated intracellular fluorescence throughout the cytoplasm. (B) A line‐scan intensity analysis across a representative cell shows that NB signals are localized between the membrane and nucleus, indicating cytoplasmic internalization. (C) US 3D reconstructions of NB‐labeled cells embedded in gelatin at different cell concentrations show concentration‐dependent increases in detectable echogenic signals, whereas unlabeled samples show minimal background signal. (D) A schematic illustrates the flow phantom setup used for dynamic US imaging of NB‐labeled cells, consisting of a peristaltic pump connected to a perfusable gelatin tunnel. (E) Bright‐field microscopy images of unlabeled and NB‐labeled macrophages show comparable cell morphology and no apparent aggregation after labeling (scale bar: 50 µm). (F) Representative B‐mode and contrast mode US images acquired under flow conditions illustrate enhanced detectability of NB‐labeled cells compared with unlabeled cells. (G) ULM‐based reconstruction of NB‐labeled cell trajectories under flow conditions reveals a laminar flow profile within the channel. The tracks are color‐coded by velocity, with the maximum velocity per pixel plotted.

To verify the intracellular localization of NB, fluorescently labeled NB were used, and confocal laser scanning microscopy was performed, followed by 3D reconstruction. As shown in Figure 3A, NB‐labeled macrophages exhibited distinct intracellular fluorescence signals distributed throughout the cytoplasmic volume. The NB signal (Coumarin 6, in green) was spatially separated from both the plasma membrane (WGA, in red) and the nucleus (Hoechst, in blue), indicating internalization rather than surface association. Line‐scan intensity analysis across representative cells further confirmed this observation (Figure 3B). The NB fluorescence peak was located between the membrane and nuclear signals, demonstrating that NB were localized within the cytoplasm. Furthermore, fluorescence quantification indicated that the NB were densely packed within the cells, with an estimated 431 ± 40 NB present per cell. Together, the 3D reconstruction, quantitative line‐scan analysis, and fluorescence quantification provide evidence for strong NB internalization, consistent with previous reports of nanoparticle uptake by cells [28].

To further evaluate whether NB‐labeled cells could be resolved in 3D, serial US scans were acquired using a motorized stage at different cell concentrations. As shown in Figure 3C,D US reconstructions revealed discrete echogenic signals corresponding to individual NB‐labeled cells. Increasing the cell concentration from 1 × 10^5^ to 1 × 10^6^ cells/mL resulted in a progressive increase in the number of detectable echoes, whereas control samples without NB labeling exhibited minimal background signal. At intermediate cell concentrations, individual cell‐associated echoes could be clearly distinguished with good spatial separation, demonstrating that NB labeling enables 3D visualization of cells with sufficient spatial resolution to resolve individual objects. Building on the static ultrasound measurements, we next explored whether NB‐labeled cells could be processed using ULM, a method previously established for MB tracking by our group [23]. Under static conditions, individual echogenic events associated with NB‐labeled cells could be successfully localized using the ULM pipeline (Movie S2), indicating that the localization step was feasible for cell‐associated signals. To further evaluate the detectability of NB‐labeled cells under dynamic conditions, US measurements were performed in a flow setup using a peristaltic pump and a gelatin phantom containing a perfusable vessel‐like tunnel (Figure 3D). Prior to US imaging, cells were examined under bright‐field microscopy. The morphology of NB‐labeled cells was comparable to that of unlabeled cells, and no apparent cell aggregation was observed following labeling (Figure 3E), indicating that NB labeling did not alter cell integrity or induce clustering. Then, cells were perfused through the flow chamber and imaged dynamically by US. As shown in Figure 3F, perfusion of unlabeled cells produced weak signals in B‐mode but no detectable contrast‐mode signal. In contrast, NB‐labeled cells generated distinct signals in both B‐mode and contrast mode, with individual echogenic events clearly visible as they traversed the flow channel (Movie S3). Finally, ULM analysis was applied to the dynamic flow data to assess whether individual NB‐labeled cells could be localized and tracked over time. Localized cell‐associated echoes were successfully tracked and reconstructed into trajectories, with velocity information extracted and color‐coded along the tracks (Figure 3G). The reconstructed trajectories revealed a laminar flow profile, with higher velocities near the center of the channel and lower velocities toward the boundaries, consistent with expected fluid dynamics in blood vessels. These results demonstrate that NB‐labeled cells can be localized, tracked, and quantitatively analyzed under flow conditions using ULM, supporting the feasibility of combining NB labeling with ULM for dynamic cell tracking.

### 2.3 US Imaging of NB‐Labeled Bone Marrow‐Derived Mononuclear Cells

**FIGURE 4:**
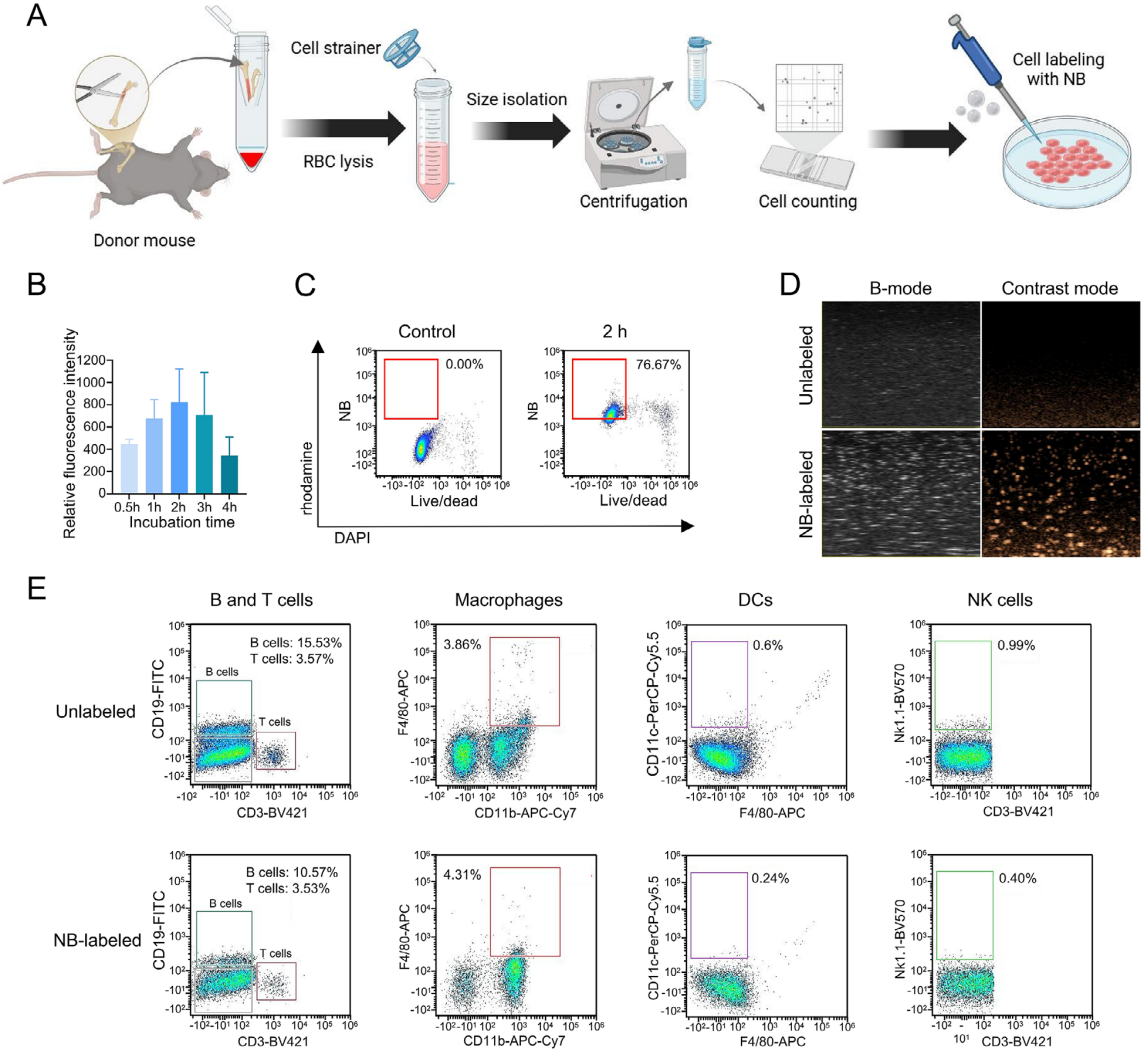
Preparation and characterization of NB‐labeled BMMC. (A) Schematic illustrating the isolation of BMMC from donor mice, including red blood cell lysis, size‐based enrichment, centrifugation, and cell counting prior to NB labeling. (B) Time‐dependent uptake of rhodamine‐labeled NB by BMMC was quantified by fluorescence measurements, showing maximal uptake after 2 h of incubation (*n = 3*). Data are shown as mean ± SD. (C) Flow cytometry analysis confirms internalization of rhodamine B‐labeled NB by viable BMMC following 2 h of incubation. (D) Representative B‐mode and contrast mode US images of unlabeled and NB‐labeled BMMC embedded in a gelatin phantom demonstrate enhanced echogenicity after NB labeling. (E) Flow cytometry–based immunophenotyping of BMMC before and after NB labeling shows preserved distributions of B cells, T cells, macrophages, dendritic cells, and natural killer cells.

Next, we replaced the J774A.1 macrophage cell line with primary bone marrow–derived mononuclear cells (BMMC) for subsequent labeling and in vivo evaluation, as these cells more closely recapitulate the cellular compositions used in therapeutic cell transplantation. BMMC were isolated from donor mice using red blood cell lysis followed by size‐based enrichment and centrifugation, as illustrated in Figure 4A. Given the heterogeneous immune cell composition of BMMC, we tested the NB labeling protocol again by varying the incubation time. Rhodamine‐loaded NB were used to enable quantitative assessment of uptake. Fluorescence measurements revealed a time‐dependent increase in NB uptake, with maximal signal observed after 2 h of incubation (Figure 4B). This finding was further confirmed by flow cytometry, which demonstrated a substantial population of rhodamine‐positive, viable BMMC after 2 h of incubation (Figure 4C), indicating efficient internalization of NB under these conditions. Furthermore, flow cytometry analysis confirmed that NB labeling did not induce BMMC aggregation (Figure S6). We next evaluated the echogenicity of NB‐labeled BMMC using gelatin phantoms. Similar to observations with J774A.1 cells, NB‐labeled BMMC generated detectable signals in both B‐mode and contrast‐mode US (Figure 4D), confirming that NB labeling confers acoustic visibility to primary immune cells. To further investigate the intracellular fate of internalized NB, we performed a fluorescence‐based excretion assay. Cells were labeled with Rhodamine‐loaded NB. Cells were washed and cultured for up to 24 h, during which the fluorescence intensity of the supernatant was monitored. As shown in Figure S7, a gradual and time‐dependent increase in supernatant fluorescence was observed, indicating that cells actively process and excrete the NB or their degradation products via exocytosis. Finally, to assess whether NB labeling alters the cellular composition of BMMC, we analyzed key immune cell subpopulations by flow cytometry after incubation with NB. As shown in Figure 4E, NB labeling preserved the physiological distribution of major immune subsets, including B cells (10.3 ± 0.3%), T cells (3.3 ± 0.2%), macrophages (4.3 ± 0.2%), dendritic cells (0.2 ± 0.1%), and natural killer cells (0.4 ± 0.2%). These proportions were comparable to those of unlabeled controls, indicating that the labeling procedure did not skew immune cell populations. To further support our claim that NB labeling does not impair the therapeutic and functional competence of the cells, we evaluated the activation capacity of T cells following NB uptake, as T cells are a major component of the mononuclear cell population and the primary effector cells in cellular immunotherapies. T cells were incubated with Rhodamine‐loaded NB, washed, and subsequently stimulated with anti‐CD3 and anti‐CD28 antibodies to mimic physiological antigen‐presenting cell engagement (Figure S8A). Flow cytometry analysis of the early activation marker CD69 revealed that while resting T cells maintained basal expression levels, a distinct subpopulation of T cells successfully internalized the NB. Crucially, upon stimulation, these NB‐labeled cells can also exhibit CD69 upregulation, evidenced by the presence of the double‐positive cell population (Rho^+^/CD69^+^) in the upper‐right quadrant (Figure S8B). This demonstrates that intracellular NB labeling does not interfere with T cell activation. Thus, our results demonstrate that NB labeling is compatible with primary BMMC, preserves key aspects of immune cell functionality, and enables US‐based detection, supporting its suitability for subsequent in vivo cell‐tracking studies.

### 2.4 In Vivo Cell Tracking Using Super‐Resolution US

**FIGURE 5:**
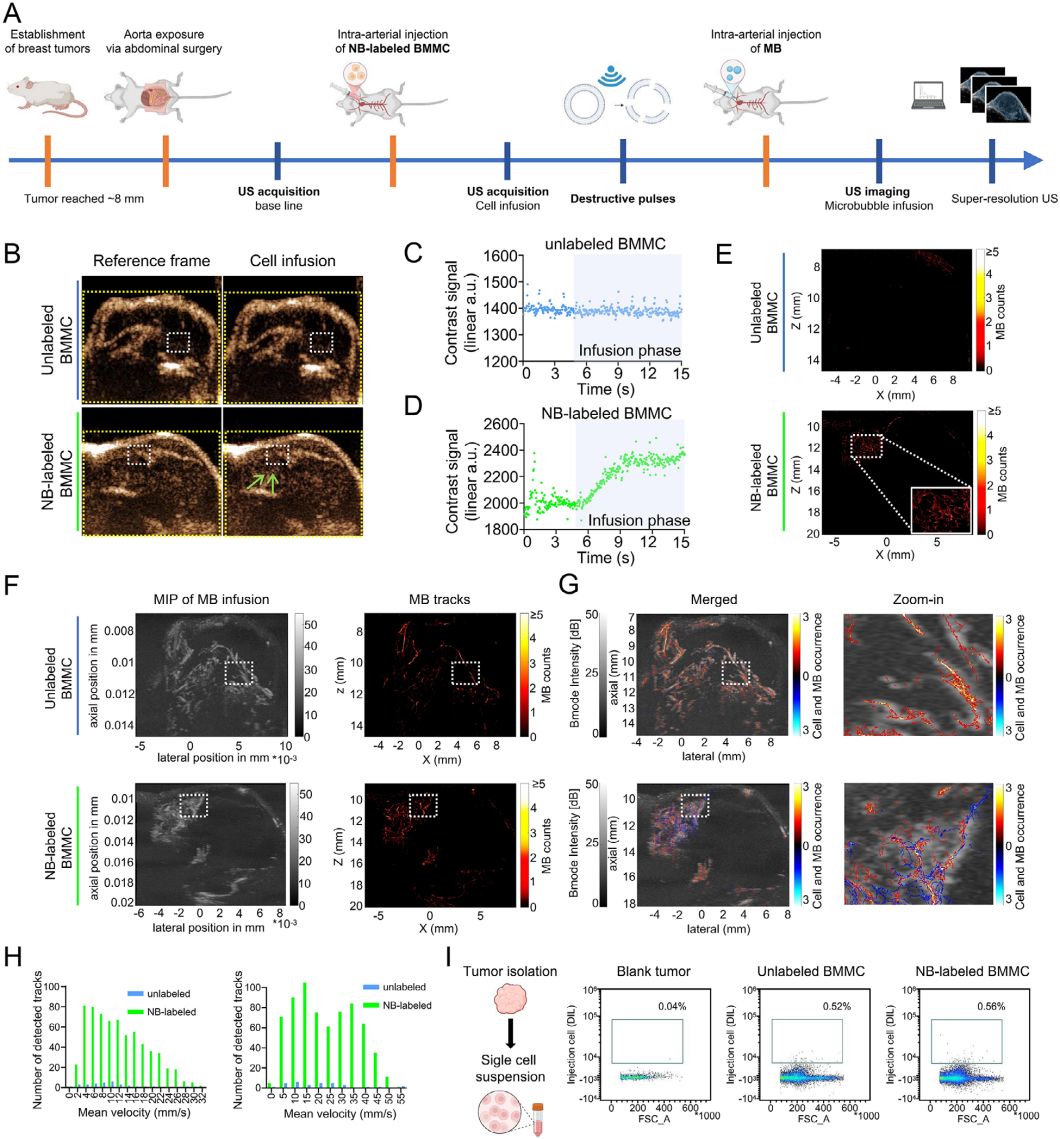
In vivo tracking of NB‐labeled BMMC in murine breast carcinomas using ULM. (A) The experimental timeline and schematic illustrate the experimental workflow of the in vivo cell‐tracking, including breast tumor establishment and US imaging steps consisting of baseline acquisition, cell injection, cell infusion recording, application of a destructive pulse, MB injection, MB infusion recording, and post‐processing with ULM. (B) Representative contrast mode US images of tumors acquired before and during infusion of unlabeled or NB‐labeled BMMC show detectable contrast enhancement only in the NB‐labeled group. The green arrows indicate the enhanced signals from NB‐labeled cells. The large yellow dashed boxes indicate the specific Regions of Interest (ROIs) selected for subsequent ULM processing displayed in panels F‐G. The two rows represent separate acquisitions from different animals, which are marked with a blue vertical line (unlabeled BMMC group) and a green vertical line (NB‐labeled BMMC group). (C) Time–intensity curves of tumor contrast signals acquired during infusion of unlabeled BMMC show no appreciable signal enhancement. (D) Time–intensity curves acquired during infusion of NB‐labeled BMMC show a significant increase in contrast signals within the tumor. (E) ULM‐based reconstruction of in vivo cell tracks shows sparse trajectories in the unlabeled group and robust cell‐associated tracks in the NB‐labeled group. (F) MIP and corresponding MB track map reconstructed from the MB infusion sequence delineate perfused tumor vasculature within the corresponding ROIs. (G) Merged overlays of the vascular MIP, MB tracks (in red), and cell tracks (in blue) show that NB‐labeled cell trajectories predominantly co‐localize with perfused vessels. White dashed boxes B, C, and G (merged images) correspond to the zoom‐in regions displayed on the right, highlighting the precise co‐localization of cell tracks within the microvasculature. (H) Distributions of mean and maximum velocities of per‐track extracted from reconstructed cell trajectories are shown for unlabeled and NB‐labeled groups. (I) Flow cytometry analysis of single‐cell suspensions prepared from excised tumors immediately after imaging confirms the presence of injected cells in both unlabeled and NB‐labeled groups.

We next investigated the feasibility of ULM‐based cell tracking in vivo using a murine breast cancer model. As an initial approach, EO771 cells were injected orthotopically into mice, and imaging experiments were initiated once tumors reached a diameter of approximately 6 mm. NB‐labeled BMMC were administered intravenously to EO771 tumor‐bearing mice. Although perfusion signals were detected in the caval veins by US (Movie S4), only minimal contrast enhancement was observed within the tumor during injection, likely due to temporary entrapment of circulating cells within the pulmonary capillary bed. However, immunofluorescence analysis of tumor tissue harvested 6 h post‐injection demonstrated that the injected cells successfully extravasated beyond the CD31‐positive vasculature and infiltrated the tumor microenvironment (Figure S9), indicating the NB‐labeled cells retain their biological ability to extravasate and infiltrate the tumor tissue. Based on these observations, subsequent experiments were performed using intra‐arterial administration to improve tumor‐associated signal detection. Here, tumors were induced by subcutaneous injection of 4T1 cells, and experiments were initiated once tumors reached a diameter of approximately 8 mm. Subsequently, mice received intra‐arterial injections of either unlabeled BMMC (*n* = 10) or NB‐labeled BMMC (*n* = 10). Intra‐arterial delivery required abdominal surgery to expose the aorta prior to cell injection (Figure 5A). US imaging was performed during cell infusion. Injection of unlabeled BMMC did not produce detectable contrast signals within the tumor region (Figure 5B,C; Movie S5). In contrast, infusion of NB‐labeled BMMC resulted in a clear increase in contrast mode US signals within tumors, indicating successful detection of circulating labeled cells (Figure 5B; Movie S6). Quantitative time–intensity analysis further confirmed a progressive enhancement of contrast signal during NB‐labeled cell infusion, followed by signal plateauing as infusion progressed (Figure 5D). To remove residual NB‐derived contrast signals, a high‐intensity destructive US pulse was subsequently applied. PBCA MB were subsequently injected, and MB infusion sequences were acquired for ULM analysis to map the tumor vasculature. ULM processing was applied to both the cell infusion and MB infusion datasets. Localized cell‐associated echoes were reconstructed into cell track maps to visualize in vivo cell trafficking. As shown in Figure 5E, NB‐labeled cells generated sufficient acoustic signals to enable localization and trajectory reconstruction, resulting in high‐quality cell track maps within the tumor volume. In contrast, only sparse and short tracks were reconstructed in the unlabeled group, likely arising from motion‐related artifacts such as respiratory movement or physiological tremor under anesthesia.

To provide anatomical context, maximum intensity projection (MIP) images were reconstructed during ULM processing from tissue‐suppressed MB infusion sequences, which delineated perfused tumor vasculature (Figure 5F). Overlaying the MIP (reference layer), MB tracks (red), and cell tracks (blue) enabled direct comparison between vascular architecture and cell trafficking (Figure 5G). While vascular maps were successfully reconstructed in both groups (Figure 5F), no cell‐associated tracks were observed in perfused vessels in the unlabeled group (Figure 5E). Conversely, in the NB‐labeled group, reconstructed cell tracks predominantly co‐localized with MB‐defined vessels and appeared as clustered trajectories aligned with vascular pathways, indicating that labeled cells primarily traveled within strongly perfused vessels (Figure 5G). Velocity information was extracted from reconstructed cell trajectories in both groups. Substantially more velocity events were detected in the NB‐labeled group than in the unlabeled group. In vessels, NB‐labeled cells moved slightly more slowly than MB (mean velocity 1.2 ± 0.6 vs. 1.5 ± 0.7 mm/s), consistent with laminar flow behavior in which larger particles exhibit reduced velocities [29]. The maximum velocity distribution of NB‐labeled cell tracks spanned distinct low‐ and high‐velocity ranges, which may reflect heterogeneous flow profiles within the tumor vasculature (Figure 5H).

To verify that injected cells reached the tumor, mice were euthanized immediately after imaging, and tumors were harvested for flow cytometry analysis. Single‐cell suspensions prepared from excised tumors were analyzed by flow cytometry with a fixed acquisition of 100 000 events per sample. This analysis confirmed the presence of injected cells in both the unlabeled group (0.88 ± 0.2%) and the NB‐labeled group (0.96 ± 0.4%; Figure 5I). Together with the ULM‐based imaging and tracking results, these findings confirm successful delivery of transplanted cells to the tumor and demonstrate the feasibility of NB‐enabled ULM for in vivo cell tracking.

## 3 Discussion and Conclusion

In this study, we established an NB‐based acoustic labeling strategy that enables ULM to track transplanted cells in vivo with single‐cell‐scale. By combining intracellular labeling with stable PBCA NB and advanced ULM processing, we achieve visualization of cell trafficking within tumors with super‐resolution spatial precision. This approach overcomes key limitations of existing cell‐tracking modalities, including insufficient sensitivity, reliance on ionizing radiation, or the need for genetic modification, and provides both spatial and dynamic information on cell transport in the tumor microenvironment using a clinically compatible imaging platform.

The selection of PBCA NB as intracellular acoustic labels is a key determinant of the performance of this system. Recent studies have questioned whether NB are intrinsically echogenic, proposing that observed US signals may instead arise from contamination by microscale bubbles or from bubble coalescence during insonation rather than from nanoscale agents themselves [30, 31]. Although these concerns are particularly relevant to lipid‐based nanoemulsion formulations, our results suggest that they do not apply universally to polymeric systems. Using a PBCA shell, we generated NB with high colloidal stability under physiological and acidic conditions, resistance to coalescence, and sufficient echogenicity. Coulter counter analysis confirmed the absence of microscale bubble contamination, while NTA and cryo‐SEM provided direct evidence of a hollow gas‐core architecture. Together, these findings support that the detected US signals originate from bona fide NB rather than secondary artifacts.

Previous studies have shown that MB can be used to engineer echogenic macrophages [32]. However, MB are inherently buoyant and prone to flotation, complicating homogeneous cell–bubble interactions and often necessitating specialized incubation setups (e.g., inverted culture systems) [33]. Beyond these technical considerations, MB–cell interactions have been shown to alter the effective buoyant properties of labeled cells [34]. Such changes in buoyancy may influence cell transport and migratory behavior in vivo. Moreover, internalization of MB via macropinocytosis imposes higher energetic and lysosomal stress on cells [35, 36]. By contrast, NB exhibit lower buoyancy, readily interact with cells under standard culture conditions, and are internalized efficiently without compromising viability at optimized doses. These properties make NB more suitable for cell‐labeling applications. From a translational perspective, PBCA NB can be produced in a lyophilized powder form before bubble reconstitution, facilitating storage, transport, and scalable manufacturing. This could be an important consideration for future clinical deployment. Moreover, it is important to take into account the fundamental physical limits of acoustic scattering at the nanoscale. In the realm of Rayleigh scattering, the scattering cross‐section of a bubble scales with the sixth power of its radius (r^13,14^) [37]. Consequently, as shown in our comparisons with matched gas volumes (Figure S3), individual free‐floating NB naturally generate weaker acoustic signals than an equivalent gas volume of micrometer‐sized MB. However, the goal of our approach is not to replace MB for conventional vascular imaging, but to enable intracellular labeling that is as physiologically unobtrusive as possible—a task that is physically difficult for buoyant MB. Ultrasonic excitation and destruction of intracellular MB can severely damage cells [38]. In addition, although individual NB scatter less than MB, robust acoustic detectability can result from collective scattering. In our study, a quantitative fluorescence analysis revealed that an average of 431 ± 40 NB accumulate densely within the highly confined volume of a single cell's cytoplasm. This dense intracellular clustering generates a significant localized acoustic impedance mismatch, allowing the cells to act as macroscopic acoustic scatterers capable of generating the distinct nonlinear signals required for ULM tracking.

Intracellular labeling through endocytosis represents another key design feature of this approach. NB uptake was achieved by simple incubation, without chemical modification, targeting ligands, or genetic manipulation. Once internalized, NB can be physiologically processed through degradation or exocytosis (Figure S7), minimizing long‐term perturbation of cell function [39, 40]. This strategy is broadly applicable to endocytosis‐competent cells and extends beyond macrophages to clinically relevant primary immune cell populations and different stem and progenitor cells. In contrast, extracellular or surface‐bound labeling strategies often require membrane modification, which may alter cell signaling, migration, or immunogenicity [41, 42]. Genetic acoustic reporters, such as gas vesicles, offer elegant biological solutions but face challenges related to transfection efficiency, regulatory complexity, and manufacturability for human studies [43, 44]. The NB‐based strategy presented here provides a non‐genetic, transient, and workflow‐compatible alternative.

Our in vivo experiments also underscore the importance of administration route for effective cell tracking. When NB‐labeled cells were initially delivered via intravenous injection, perfusion signals could be detected in the caval veins. However, weak contrast enhancement was observed within the tumor during injection. This observation is consistent with previous reports showing that intravenous injection frequently results in pulmonary trapping of administered cells, limiting delivery to distal target sites such as tumors [45, 46, 47, 48]. Guided by these findings, we adopted an intra‐arterial delivery strategy, which increased the number of cells reaching the tumor and enabled US detection. Using this approach, NB‐labeled BMMC generated visible contrast enhancement during infusion, whereas unlabeled cells remained largely undetectable. These findings highlight that successful in vivo cell tracking depends on both an effective labeling strategy and an optimized delivery route.

A major advance of this work is the integration of intracellular NB labeling with ULM. To our knowledge, this is the first demonstration of ULM applied to track transplanted cells in vivo. Beyond detecting cell presence, ULM enabled reconstruction of individual cell trajectories and extraction of velocity information with micrometer‐scale resolution. By combining cell tracks with MB‐derived vascular maps, we were able to distinguish vessels that were perfused by circulating cells from those that were not. It is important to note that within the finite acquisition windows feasible for in vivo imaging, ULM provides a sparse sampling of the total vasculature. Based on established zero‐inflated Poisson (ZIP) saturation models, achieving a 100% complete super‐resolution track map is statistically challenging [49]. In this study, the degree of microvascular reconstruction (DOR) for the MB sequence is estimated to be between 30% and 47%. Consequently, the reconstructed MB tracks represent a stochastic subset of the vascular network, which explains why some cell trajectories do not perfectly overlap with the detected MB tracks. Despite this sparsity, these maps provide critical information regarding cell localization and hemodynamics. Overlapping tracks or equivalent propagation speeds between NB‐labeled cells and MB confirm intraluminal transit. Furthermore, a localized decrease in cell velocity may indicate the initiation of extravasation, such as rolling along the endothelium. Conversely, if a cell ceases movement entirely, it has likely attached to the vessel wall or completed extravasation. If a cell track appears where no corresponding MB track exists, confidently distinguishing between intra‐ and extravascular localization remains difficult with current tracking alone. In the future, integrating multiple ULM features alongside MIP and Doppler data through advanced simulations may enable the generation of more comprehensive and reliable vascular maps, further supporting the precise anatomical localization of transplanted cells within the tissue.

ULM has traditionally been developed as a tool for microvascular imaging, yet its broader clinical adoption has been limited by workflow complexity and reliance on histology as a reference standard [23, 50, 51]. By extending ULM from vessel mapping to dynamic cell tracking, our work substantially expands its application space. Integrating NB‐based cell labeling with ULM offers a non‐ionizing and spatially precise method to evaluate cell‐based therapies. This capability addresses critical unmet clinical needs by moving beyond static biodistribution to functionally map cell‐vascular interactions in real‐time. For example, in regenerative medicine for ischemic stroke, therapeutic cells are frequently delivered intra‐arterially to bypass pulmonary trapping [45]. While conventional MRI can detect bulk accumulation, our NB‐ULM platform could dynamically verify whether these cells successfully navigate the cerebral microcirculation to reach the ischemic penumbra. Furthermore, in the context of immuno‐oncology, a major barrier to CAR‐T cell therapy in solid tumors is heterogeneous vascular delivery. NB‐ULM could definitively map which specific tumor areas permit CAR‐T cell transit and which regions remain inaccessible due to abnormal vasculature. Furthermore, by comparing velocities of NB‐labelled cells and MB, insights into the interaction of cells with the vascular walls as a starting point of extravasation can be provided, which is important for all applications mentioned above. These advances may accelerate the clinical translation of ULM by providing actionable information on cell delivery, distribution, and dynamics that is difficult to obtain with existing imaging techniques.

At the same time, several technical challenges remain. The US data in this study were acquired using a preclinical high‐frequency system, which, while offering high spatial resolution, also introduces elevated background noise and limited penetration depth [52]. Furthermore, operating in non‐linear contrast mode on commercial US systems inherently restricts temporal resolution. In this study, data were acquired at ∼23 fps. While such low frame rates can limit track density compared to ultrafast plane‐wave imaging, our post‐processing pipeline utilizes a robust Markov Chain Monte Carlo Data Association (MCMCDA) algorithm. This motion‐model tracking approach correctly bridges spatial gaps between frames and has been previously validated by our group to successfully reconstruct super‐resolution microvascular maps in human patients at frame rates as low as 10 Hz [51]. Although our platform successfully tracks the intravascular motion of NB‐labeled cells, a limitation of the current study is the difficulty of tracking these cells longitudinally once they extravasate into the tumor microenvironment. Ex vivo histological analysis (Figure S9) confirmed that the cells successfully exit the vasculature and infiltrate the tumor tissue six hours post‐injection. However, once localized within the tissue, these cells become largely stationary. Current ULM algorithms rely heavily on the movement of scatterers to separate them from the static tissue background. Therefore, these stationary cells are difficult to isolate acoustically. Achieving longitudinal in vivo tracking of extravasated cells will require developing advanced, motion‐independent, nonlinear filtering algorithms that can robustly suppress tissue clutter. Another limitation relates to the intracellular fate of NB. Internalized NB may undergo physiological exocytosis or degradation over time, which may limit their suitability for long‐term cell tracking [53, 54]. In this context, genetically encoded gas vesicle reporters offer an alternative strategy for sustained tracking, as contrast can be maintained through continuous gene expression and inheritance by proliferating cells [43, 44]. However, the clinical translation of such approaches remains challenging due to the requirement for genetic modification and the associated regulatory and safety considerations.

In summary, our results demonstrate that stable PBCA NB enables reliable intracellular acoustic labeling and, when combined with ULM, allows super‐resolution tracking of transplanted cells in vivo. This platform addresses key limitations of current cell‐tracking approaches and provides a foundation for future studies aimed at monitoring, optimizing, and personalizing cell‐based therapies.

## 4 Experimental Section

### 4.1 Synthesis of PBCA Polymers

Poly(butyl cyanoacrylate) (PBCA) was synthesized via anionic polymerization following a previously reported protocol with minor modifications [55]. Briefly, 3 mL of butyl cyanoacrylate (BCA) monomer was added to 10 mL of tetrahydrofuran (THF) containing 1 mm trifluoroacetic acid in a round‐bottom flask. Separately, 12.8 mg of sodium methoxide (NaOMe) was dissolved in 200 µL of ultrapure water to prepare the initiator solution. The initiator was added dropwise to the monomer solution under continuous stirring (400 rpm) and allowed to react for 180 min at room‐temperature. The polymerization was quenched by precipitation into 45 mL of 0.1 m hydrochloric acid (HCl). The resulting polymer was collected by centrifugation (3000 rpm, 5 min), and the supernatant was carefully removed. The obtained PBCA precipitate was snap‐frozen in liquid nitrogen and lyophilized overnight under reduced pressure (0.01 mbar). The successful polymerization of BCA was verified by gel permeation chromatography (GPC). Samples were prepared by dissolving PBCA in dimethylformamide (DMF) containing 10 mm LiCl at a concentration of 5 mg/mL. GPC analysis was performed using a PLgel 3 µm MIXED‐E column (300 × 7.5 mm; Agilent Technologies, Waldbronn, Germany) with DMF containing 10 mm LiCl as the eluent at a flow rate of 0.5 mL min^−^^1^ and a column temperature of 55°C. Poly(ethylene glycol) standards (Agilent Technologies, Waldbronn, Germany) were used for calibration according to the manufacturer's instructions. Data acquisition and processing were conducted using Cirrus GPC software (Agilent Technologies, Waldbronn, Germany).

### 4.2 Preparation of PBCA NB

PBCA NB were prepared using a water‐in‐oil‐in‐water (W/O/W) double‐emulsion solvent evaporation method followed by freeze‐drying to generate the hollow gas‐filled structure. Briefly, PBCA polymer (100 mg) was dissolved in 2 mL of methylene chloride (DCM) to form the organic phase. Subsequently, 0.2 mL of ultrapure water was added, and the mixture was emulsified by probe sonication at 35% amplitude for 2 min in an ice bath (Branson Ultrasonics, Danbury, CT, USA) to obtain the primary (W/O) emulsion. This primary emulsion was immediately poured into 10 mL of 1% (w/v) polyvinyl alcohol (PVA) aqueous solution and sonicated for an additional 3 min to generate the secondary (W/O/W) emulsion. The resulting emulsion was stirred overnight at room‐temperature to allow complete evaporation of DCM. The nanoparticles were purified by dialysis (molecular weight cut‐off: 6–8 kDa) against ultrapure water for 24 h, followed by freeze‐drying to remove the internal aqueous phase and generate a hollow gas‐filled structure, and then stored at 4°C until use. For fluorescent labeling, Coumarin 6‐ or Rhodamine B–loaded PBCA nanobubbles were prepared using the same procedure, except that 0.2 mL of Coumarin 6 (0.2 mg/mL in DCM) or Rhodamine B (1 mg/mL in DCM) solution was added to the organic phase during the formation of the primary (W/O) emulsion, instead of ultrapure water.

### 4.3 Characterization of PBCA NB

The morphology and size of the nanobubbles were characterized by scanning electron microscopy (SEM; Hitachi SU9000, Mannheim, Germany), transmission electron microscopy (LEO 906, Carl Zeiss, Oberkochen, Germany), and cryogenic scanning electron microscopy (Cryo‐SEM; Hitachi FE‐SEM 4800, Krefeld, Germany). For SEM and TEM imaging, 10 µL of the nanobubble suspension was deposited onto silicon wafers or carbon‐coated copper grids and air‐dried. For SEM observation, the samples were further coated with a thin conductive carbon layer (∼3 nm). Cryo‐SEM imaging was performed after rapid freezing of sample droplets on the specimen holder, followed by controlled sublimation to remove surface ice for the preservation of the native hydrated morphology. The hydrodynamic diameter and size distribution were further measured by dynamic light scattering (Zetasizer Nano ZS, Malvern Instruments, UK) and nanoparticle tracking analysis (NanoSight NS300, Malvern Instruments, UK). Measurements were conducted at 25°C after appropriate dilution in ultrapure water, and each sample was analyzed in triplicate.

### 4.4 In Vitro US Phantom Imaging

Custom‐made gelatin‐based phantoms were used to analyze the echogenicity of PBCA‐NB. The VEVO 3100 US system, incorporating a linear MX‐250 transducer (FUJIFILM VisualSonics, Toronto, Canada), was employed for US imaging. NB was diluted with 2 mL of 2% w/v gelatin solution, and the mixture was embedded in a 10% w/v gelatin matrix. The transducer was fixed vertically above the phantom with a focal depth of 10 mm. US imaging was performed in non‐linear contrast mode at 18 MHz frequency, 4% power and a mechanical index of 0.03. The peak negative pressure at this setting is estimated to be in the range of 100 kPa, with spatial peak temporal average intensity remaining below 29 mW cm^−2^ [56]. To quantify the acoustic intensity, a region of interest (ROI) was drawn within the sample‐loaded gelatin, and acoustic intensities were quantified using the VevoLAB software version 3.2 (FUJIFILM VisualSonics, Toronto, Canada).

### 4.5 Cell Labeling With NB

The J774A.1 macrophage cell line was obtained from the American Type Culture Collection (ATCC; Manassas, VA, USA) and cultured in low‐glucose DMEM (Thermo Fisher Scientific, Darmstadt, Germany) supplemented with 10% heat‐inactivated fetal bovine serum and 1% penicillin–streptomycin at 37°C in a humidified incubator with 5% CO_2_. For NB labeling, 2 × 10^6^ cells were seeded in each well of a six‐well plate. PBCA‐NB were added at NB‐to‐cell ratios of 10 000:1, 50 000:1, or 100 000:1, followed by incubation for 2 h. Then, the cells were washed three times with phosphate‐buffered saline (PBS) to remove non‐internalized NB. The NB‐labeled cells were subsequently maintained on ice prior to further characterization.

### 4.6 US Imaging of the NB‐Labeled Cells In Vitro

The echogenicity of NB‐labeled cells under static conditions was assessed using the gelatin phantom described above. Briefly, the labeled cells were suspended in 2% (w/v) gelatin, placed in the sample holder, and imaged using the same US parameters as described previously. To evaluate the clinical translatability and acoustic stability of NB‐labeled cells at lower frequencies, in vitro imaging was also performed using a clinical ultrasound scanner (Aplio i800, Canon Medical Systems, Otawara, Japan) equipped with a clinical transducer (I8CX1). Images were acquired in nonlinear contrast mode using a center transmit frequency of 4 MHz and a low mechanical index (MI = 0.1).

To evaluate echogenicity under flow conditions, a custom gelatin phantom was constructed as described previously [57]. A 3D‐printed chamber with inlet and outlet ports, each with an inner diameter of 3 mm, was filled with 10% (w/v) gelatin. A cylindrical insert of matching diameter was positioned horizontally to form a tunnel connecting the inlet and outlet. After the gelatin had solidified, the insert was gently removed to create a perfusable channel. The ultrasound transducer was positioned above the channel, which was flushed with PBS using a peristaltic pump until a clear lumen was visible on US. NB‐labeled cells were then infused through the tunnel at a flow rate of 0.25 mL/min, and their passage was recorded in real‐time.

### 4.7 NB Uptake by Bone Marrow‐Derived Mononuclear Cells

All animal procedures were approved by the State Office for Nature, Environment and Consumer Protection (LANUV), North Rhine‐Westphalia, Germany. To isolate bone marrow‐derived mononuclear cells, 8‐week‐old female C57BL/6 wild‐type mice (*n* = 5) were euthanized by cervical dislocation under anesthesia with 5% isoflurane (FORENE, AbbVie AG, Ludwigshafen, Germany). Both femurs and tibias were aseptically harvested and cleaned of surrounding muscle and connective tissue. After removal of the epiphyses, the bones were placed cut‐side down into 0.5 mL nested microcentrifuge tubes (Sarstedt, Nümbrecht, Germany) inserted into 1.5 mL collection tubes and centrifuged at 5000 rpm for 1 min to collect the bone marrow. The cell pellet was resuspended in ice‐cold RPMI medium supplemented with 10% fetal bovine serum (FBS), gently triturated, and filtered through a 40 µm cell strainer to obtain a single‐cell suspension. Red blood cells were lysed by incubating the suspension with 1× RBC lysis buffer (Thermo Fisher Scientific, MA, USA) for 2 min at room‐temperature with gentle agitation, followed by quenching with excess RPMI + 10% FBS and centrifugation at 400 × g for 5 min at 4°C. Cells were washed with RPMI containing 10% FBS, filtered again through a 40 µm strainer, and counted using a hemocytometer with trypan blue exclusion.

For NB uptake studies, isolated cells were resuspended in complete medium (RPMI 1640 supplemented with 10% fetal bovine serum and 1% penicillin–streptomycin) at a density of 1 × 10^6^ cells/mL and incubated with rhodamine‐loaded PBCA‐NB at an NB‐to‐cell ratio of 10^5^:1. Uptake was assessed at 0, 0.5, 1, 2, and 4 h by flow cytometry analysis. At each time point, cells were collected and kept on ice, then washed three times with cold FACS buffer (PBS supplemented with 5% bovine serum albumin (BSA) and 2 mm EDTA), and stained with DAPI to exclude dead cells in gating of flow cytometry analysis. Flow cytometry was performed on a Sony SA3800 cytometer (Sony Biotechnology, CA, USA), determining internalization of NB by isolated bone marrow cells through detecting rhodamine fluorescence of cells. A minimum of 10 000 live, single cells per sample were acquired, and NB uptake was quantified as the percentage of rhodamine‐labeled NB‐positive cells and the median fluorescence intensity.

### 4.8 In Vivo Cell Tracking

All in vivo cell‐tracking experiments were approved by the State Office for Nature, Environment and Consumer Protection (LANUV), North Rhine–Westphalia, Germany (Approval No. 2025‐187‐Grundantrag). Female BALB/c mice (10–20 weeks old; Janvier Labs, France) were housed under specific pathogen‐free conditions in groups of 3–5 animals in individually ventilated type II long cages (Tecniplast, Germany) with spruce granulate bedding. Housing rooms were maintained at 20°C–24°C and 45%–65% humidity, with ad libitum access to water and standard laboratory chow. Breast tumors were induced by subcutaneous injection of 4T1 cells (4 × 10^4^ cells in 50 µL PBS) into the lower right dorsal region (*n* = 20), allowing direct access for US imaging. Tumor growth was monitored by caliper measurements. Once tumors reached a diameter of approximately 8 mm, mice were randomly assigned to receive either NB‐labeled cells (*n* = 10) or unlabeled control cells (*n* = 10). Mice received carprofen (25 mg/kg) in the drinking water starting 24 h before imaging. All procedures were performed under inhalation anesthesia with 2% isoflurane.

BMMC were isolated from syngeneic donor mice (BALB/c, *n* = 5) as described above. For tracking experiments, both NB‐labeled and control cells were stained with CellTracker CM‐DiI (Thermo Fisher Scientific) according to the manufacturer's instructions. Cells were washed, counted, and resuspended in sterile 0.9% saline at a concentration of 1 × 10^6^ cells per 100 µL.

Mice were positioned supine on a temperature‐controlled platform and maintained under 1.5% isoflurane anesthesia. Local analgesia was achieved by subcutaneous injection of bupivacaine (2 mg/kg, 0.25% in 0.9% saline) into the abdominal region. After confirmation of analgesia by a negative reflex test, abdominal hair was clipped, and the tumor‐bearing flank was depilated. Following abdominal disinfection, a 1.5–2 cm midline laparotomy was performed, and the intestine was exteriorized onto a pre‐warmed, saline‐moistened drape. The abdominal aorta was exposed below the renal artery bifurcation under stereomicroscopic guidance. Two ligatures were placed, and the aorta was punctured between them using a 25G cannula for catheter insertion. A mouse arterial catheter was advanced to the aortic arch, the insertion site sealed with tissue adhesive, and blood flow restored. The catheter was secured with agarose gel, the abdominal cavity filled with warm sterile saline, and the skin temporarily closed with interrupted sutures.

Under continued inhalation anesthesia, mice were positioned laterally, and the US probe was placed over the tumor. Either NB‐labeled or control cells (1 × 10^6^ cells in 100 µL saline) were injected via the arterial catheter over 1 min, with continuous monitoring of anesthesia and vital signs.

Real‐time US imaging was performed using a VEVO 3100 system (FUJIFILM Visualsonics, Toronto, Canada) equipped with an MX‐250 linear transducer operating in nonlinear contrast mode (center frequency, 18 MHz; output power, 4%; dynamic range, 40 dB; frame rate, 20–23 fps). Imaging sequences consisted of: (i) baseline acquisition before cell injection; (ii) cell infusion phase, during which imaging continued until circulating cell‐associated signals plateaued or declined; (iii) burst phase, applying a destructive ultrasound pulse (100% power) to eliminate NB‐derived signals; and (iv) microbubble infusion phase, in which PBCA MB (SonoMAC, Aachen, Germany; 2.5 × 10^8^ bubbles kg^−^^1^) were administered via the same catheter, followed by contrast‐enhanced imaging to visualize tumor vasculature.

Immediately after imaging, mice were euthanized by cervical dislocation under anesthesia. Tumors were excised and placed in ice‐cold RPMI 1640 medium supplemented with 10% fetal bovine serum for downstream flow cytometry analyses.

In vivo cell tracking following intravenous cell administration was performed in a separate tumor model (Approval No. 81‐02.04.2023.A058). Here, breast tumors were induced in female C57BL/6 mice (10–12 weeks old) by orthotopic injection of EO771 cells (2.5 × 10^5^ cells in 50 µL PBS; *n* = 20). Once tumors reached a diameter of approximately 6 mm, mice were randomized to receive either NB‐labeled cells (*n* = 10) or control cells (*n* = 10). BMMC were isolated from syngeneic donor mice (*n* = 5). Cells (1 × 10^6^ in 100 µL saline) were injected intravenously via the tail vein using a 27G needle over 1 min. US imaging and post‐imaging procedures were performed as described above.

### 4.9 Flow Cytometry Analysis for Transplanted Cells

Tumors were excised and transferred to ice‐cold RPMI 1640 medium supplemented with 10% FBS. Tumor tissues were minced with sterile surgical scissors and enzymatically digested in RPMI 1640 containing collagenase type IV (1 mg/mL; Sigma–Aldrich, St. Louis, MO, USA), hyaluronidase (0.5 mg/mL; Sigma–Aldrich), and DNase I (50 U/mL; Merck, Darmstadt, Germany) at 37°C for 30 min under gentle agitation. The resulting suspension was triturated and passed through a 40 µm cell strainer to obtain a single‐cell suspension. Cells were centrifuged (400 × g, 5 min, 4°C), treated with 1× RBC lysis buffer to remove erythrocytes, and resuspended in FACS buffer. The suspension was filtered again through a 40 µm cell strainer to eliminate aggregates.

For antibody staining, isolated tumor cells were incubated on ice with the following fluorescence‐conjugated antibodies, purchased from BioLegend, San Diego, CA, USA, for 30 min: B220–PE‐Cy7 (B cells), CD3–BV421 (T cells), F4/80–APC and CD11b–BV605 (macrophages), and CD11c–PerCP‐Cy5.5 (dendritic cells). DAPI was subsequently stained for 2 min before acquisition to exclude non‐viable cells. After staining with antibodies, samples were washed twice with FACS buffer and resuspended in FACS buffer for flow cytometry analysis. To account for tumor‐associated autofluorescence, untreated 4T1 tumors from BALB/c mice (*n* = 1) were included as blank controls to define baseline fluorescence. Data were analyzed using SA3800 spectrum analyzer software (version 2.0, CA, USA).

### 4.10 Ultrasound Localization Microscopy

To obtain the cell trajectory maps, ULM was performed on IQ data from B‐mode measurements for tumor imaging sequences and of nonlinear contrast mode for in vitro measurement using MATLAB (MathWorks, Natick, MA, USA) [58]. A reference frame was determined, which corresponds to the frame with the highest normalized cross‐correlation to all other frames. This frame served as the coordinate system for the final images. ULM processing was applied to manually defined regions of interest, including the tumor for in vivo and the flow channel region for in vitro data. For in vivo measurements, the MATLAB function imregdemons was used to apply motion correction to compensate for probe drift and physiological motion. The extraction of moving contrast signals in B‐mode was achieved through the implementation of singular value decomposition (SVD) filtering, a process that effectively removed tissue clutter. Individual contrast signals were localized using a Gaussian matched filter and tracked using the Markov Chain Monte Carlo Data Association (MCMCDA) algorithm [59]. The same ULM processing pipeline was applied to the microbubble (MB) infusion sequence, enabling reconstruction of MB trajectories representing the tumor vasculature. For this, motion correction was performed in relation to the reference frame of the cell injection measurement. Trajectories with a length of ≥ 3 localization events per track were reconstructed using a Rauch‐Tung‐Striebel smoother approach on a grid with a pixel resolution of 8 µm [60]. Final in vivo super‐resolution maps were generated by overlaying the reconstructed NB‐labeled cell tracks, MB‐derived vascular trajectories, and the corresponding B‐mode anatomical images (MIP), yielding super‐resolution cell‐tracking images.

### 4.11 Statistical Analysis

Data are presented as means ± standard deviations (SD). Statistical differences between two groups were assessed using an unpaired two‐tailed Student's *t*‐test. Comparisons involving more than two groups were evaluated using one‐way analysis of variance (ANOVA) followed by Tukey's post hoc test. All analyses were performed using GraphPad Prism 10 (GraphPad Software, San Diego, CA, USA). Statistical significance was assumed at ^*^ *p* < 0.05, ^**^ *p* < 0.01, ^***^ *p* < 0.001, as indicated in the corresponding figure legends.

## Author Contributions

J.C., A.R., and F.K. designed the experiments. J.C., R.B. and J.F. optimized the synthesis of NB formulation. J.C. and X.W. performed the in vitro experiments. M.H. offered bones from donor mice. B.W. and M.R. prepared the single‐cell suspension of mouse tissue. J.C., B.W., H.F., Y.W., H.C., and Y.S. acquired the flow cytometry data and designed the gating methods. A.R., A.K., Q.P., and T.La. contributed to the ethics declarations of in vivo experiments. J.C. and A.R. performed the in vivo experiments and acquired the ultrasound data. T.Li., G.S., and F.K. developed ULM methods for cell tracking. J.C. wrote the first draft of the manuscript. All the authors contributed to reviewing and editing the manuscript.

## Conflicts of Interest

A.R., T.La., and F.K. are co‐owners and founders of the SonoMAC GmbH. F.K. is advisor of FUJIFILM VISUALSONICS and BRACCO.

## Supporting information




**Supporting File 1**: adma73639‐sup‐0001‐SuppMat.docx.

**Supporting File 2**: adma73639‐sup‐0002‐MovieS1.avi.

**Supporting File 3**: adma73639‐sup‐0003‐MovieS2.avi.

**Supporting File 4**: adma73639‐sup‐0004‐MovieS3.mp4.

**Supporting File 5**: adma73639‐sup‐0005‐MovieS4.mp4.

**Supporting File 6**: adma73639‐sup‐0006‐MovieS5.mp4.

**Supporting File 7**: adma73639‐sup‐0007‐MovieS6.mp4.

## Data Availability

The data that support the findings of this study are available from the corresponding author upon reasonable request.
