# Supplementary material for: Super‐Resolution Ultrasound Based Cell Tracking With Polymeric Nanobubbles
Source: Adv Mater. 2026 Jun 6;38(39):e73639. doi: 10.1002/adma.73639 (PMC13361165; doi:10.1002/adma.73639)
Supplement: Supplementary file 1 — Supporting File 1: adma73639‐sup‐0001‐SuppMat.docx. [file ADMA-38-e73639-s005.docx]

**– SUPPORTING INFORMATION –**

**Super-resolution Ultrasound Based Cell Tracking with Polymeric Nanobubbles**

***Junlin Chen^1^, Xiaoyu Wang^1^, Jilin Fan^2,3^, Bi Wang^1^, Hanghang Fang^1^, Yurui Wang^1^, Hao Cui^1^, Mohammad Roufarshbaf^1^, Ekaterina Savina^1^, Alexandra Valeske^1^, Quim Peña^1^, Yang Shi^1^, Andreas Herrmann^2,3^, Twan Lammers^1^, Mathias Hornef^4^, Roman Barmin^1^, Thomas Lisson^5^, Georg Schmitz^5^, Anne Rix^1^, Fabian Kiessling^1^****

*^1^Institute for Experimental Molecular Imaging, RWTH Aachen University, Aachen 52074, Germany*

*^2^* *Institute of Technical and Macromolecular Chemistry, RWTH Aachen University, 52074, Aachen, Germany*

*^3^DWI – Leibniz-Institute for Interactive Materials, Aachen, 52056, Germany.*

*^4^Institute of Medical Microbiology, Rheinisch-Westfälische Technische Hochschule Aachen University Hospital, Aachen 52074, Germany*

*^5^Chair for Medical Engineering, Ruhr University Bochum, Bochum, Germany*

*Corresponding author: fkiessling@ukaachen.de*

**Table of Contents**

[Supplementary Methods 3](#_Toc229483857)

[Figure S1 | Acoustic stability of NB during continuous US imaging 5](#_Toc229483858)

[Figure S2 | Acoustic characterization of isolated PBCA NB 6](#_Toc229483859)

[Figure S3 | Ultrasound echogenicity of PBCA NB and MB at matched gas volumes 6](#_Toc229483860)

[Figure S4 | Viability of NB-labeled macrophages after a destructive ultrasound pulse measured by the XTT assay. 7](#_Toc229483861)

[Figure S5 | In vitro US imaging of NB-labeled cells using preclinical and clinical systems 7](#_Toc229483862)

[Figure S6 | Evaluation of cell aggregation following NB labeling using flow cytometry. 8](#_Toc229483863)

[Figure S7 | Cellular excretion of internalized PBCA NB 8](#_Toc229483864)

[Figure S8 | Functional activation of T cells following intracellular NB labeling 9](#_Toc229483865)

[Figure S9 | Intravenously injected cells successfully extravasate into the tumor microenvironment 10](#_Toc229483866)

[Movie S1 | Nanoparticle tracking analysis of PBCA NB 11](#_Toc229483867)

[Movie S2 | ULM of 3D scanning sequence of NB-labeled cells under static conditions. 11](#_Toc229483868)

[Movie S3 | Dynamic US imaging of NB labeled cells under flow 11](#_Toc229483869)

[Movie S4 | US imaging of the liver during intravenous injection of NB-labeled BMMC. 11](#_Toc229483870)

[Movie S5 | US contrast-mode video of a subcutaneous tumor during infusion of unlabeled BMMC 11](#_Toc229483871)

[Movie S6 | US contrast-mode video of a subcutaneous tumor during infusion of NB-labeled BMMC 11](#_Toc229483872)

# Supplementary Methods

**T cell priming assay**

Naïve T cells were isolated from wild type mice (3 months old) using magnetic negative selection approach. Briefly, spleens were collected and dissociated through a 40 μm cell strainer to obtain single-cell suspensions. The cell suspension was centrifuged at 400 g for 5 min, and resuspended in MACS buffer (PBS containing 0.5% BSA and 2 mM EDTA). Cells were incubated with a biotin-conjugated antibody cocktail targeting non-T cells for 5 min at 4 °C, followed by incubation with anti-biotin microbeads for 10 min at 4 °C. After washing, the suspension was loaded onto an LS column placed in a magnetic field. The flow-through fraction containing unlabeled cells was collected as the enriched naïve T cell population, whereas magnetically labeled non-T cells were retained in the column. Purified T cells were washed, counted by trypan blue exclusion. Finaly, the isolated T cells were resuspended in complete RPMI-1640 medium for further experiment. Mouse T cell isolation kit, and the supplements, including LS column, BSA stock solution and rinsing solution, were from MACS Miltenyi Biotec (Bergisch Gladbach, Germany).

Purified naïve T cells were seeded into a 24-well plate at a density of 1.0 × 10⁶ cells per well. The cells were co-cultured with NB with a 50000:1 ratio of NB to cells for 2 hours, followed by three washes with cold MACS buffer. Prior to use, Dynabeads® Mouse T-Activator CD3/CD28 beads were prepared as follows: the beads were first resuspended by vortexing for 30 seconds and then rotated for 5 minutes. The desired volume of beads was transferred to a tube, mixed with an equal volume of buffer and vortexed for 5 seconds. The tube was placed on a magnetic rack for 1 minute, and the supernatant was discarded. The beads were then removed from the magnet and resuspended in culture medium to the same volume as the initial bead volume. Next, NB treated T cells were resuspended in 100 µL of medium per well in a 24-well plate, and 25 µL of pre-washed Dynabeads® were added to achieve a bead-to-cell ratio of 1:1. The cells were incubated at 37°C in a humidified CO₂ incubator for 3 days. After activation, magnetic beads were removed by placing the tube on a magnet for 1-2 minutes. The supernatant containing the cells was transferred to a new tube. Cells were then washed with cold MACS buffer and stained with FITC-conjugated anti-CD69 antibody to assess activation. DAPI was used to evaluate cell viability. Staining was performed for 15 minutes at 4°C in the dark. Finally, the cells were analyzed by flow cytometry using Sony SA3800 instrument. Dynabeads® Mouse T-Activator CD3/CD28 were purchased from Thermo Fisher Scientific (Darmstadt, Germany).

**Fluorescence-based quantification assays**

To quantify the average number of internalized PBCA NB per cell, a fluorescence-based calibration assay was established. First, a standard curve was generated by measuring the fluorescence intensity of Rhodamine B-loaded NB suspensions at serially diluted, known particle concentrations (10^7^~10^9^ NB/mL). For cellular quantification, cells were incubated with the Rhodamine B-loaded NB for 2 hours, thoroughly washed with PBS to remove non-internalized NB. The fluorescence intensity of the cell suspension was measured using a TECAN Infinite M200 Pro microplate reader (Excitation: 550 nm, Emission: 580 nm). The total number of internalized NB was calculated by interpolating the suspension fluorescence against the standard curve. This total value was then divided by the cell count to determine the average number of NBs packed within a single cell's cytoplasm. To evaluate the excretion of internalized NB, the NB-label cells were then cultured for 24 hours. At designated time points (0, 1, 3, 8, and 24 h), supernatant samples were collected, and their fluorescence intensity was measured. The percentage increase in fluorescence intensity was calculated using the following equation: The percentage increase in fluorescence intensity at each time point was calculated as following: FI_Increase_ (%) = [(FI_time point_ - FI_0h_) / FI_0h_] * 100%.

**Histological analysis**

Following ultrasound imaging, tumors were resected, embedded in Tissue-Tek O.C.T. compound (Sakura Finetek Europe, Alphen aan den Rijn, Netherlands), snap-frozen, and stored at −80 °C until cryosectioning. Immunofluorescence staining was performed on 8 μm-thick cryosections. The tumor sections were fixed in 80% methanol for 5 min at 4 °C, followed by treatment with acetone at −20 °C for 2 min. After fixation, the sections were washed three times with PBS and incubated overnight at 4 °C with a rat anti-CD31 monoclonal primary antibody (1:20; Dianova, Hamburg, Germany). Thereafter, the sections were washed with PBS and stained with a cyanine 3-conjugated donkey anti-rat IgG (H+L) secondary antibody (1:500; Dianova, Hamburg, Germany). Following this, sections were incubated in 4′,6-diamidino-2-phenylindole (DAPI, Thermo Fisher Scientific, Schwerte, Germany) to stain and visualize the nuclei. The sections were visualized using a fluorescence microscope (Axio Imager M2, Carl Zeiss, Oberkochen, Germany).

**
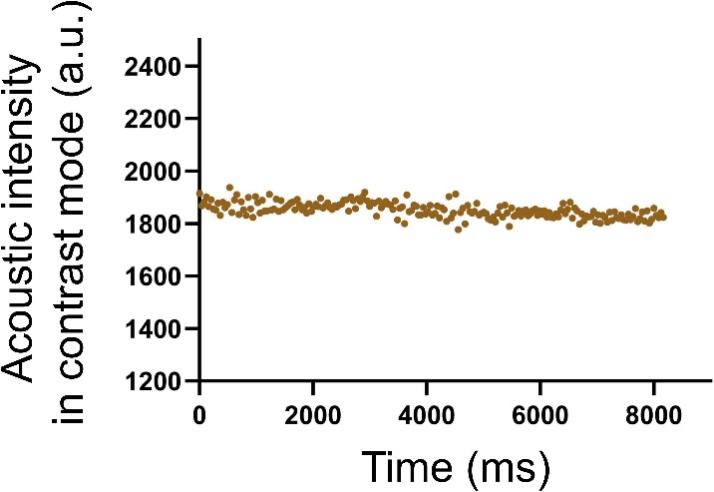
**

Figure S1 | Acoustic stability of NB during continuous US imaging. Contrast mode acoustic intensity (a.u.) of NB embedded in a gelatin phantom, recorded over a continuous US acquisition. The absence of signal decay over time indicates that the standard imaging sequence is non-destructive and allows for stable, long-term NB tracking.


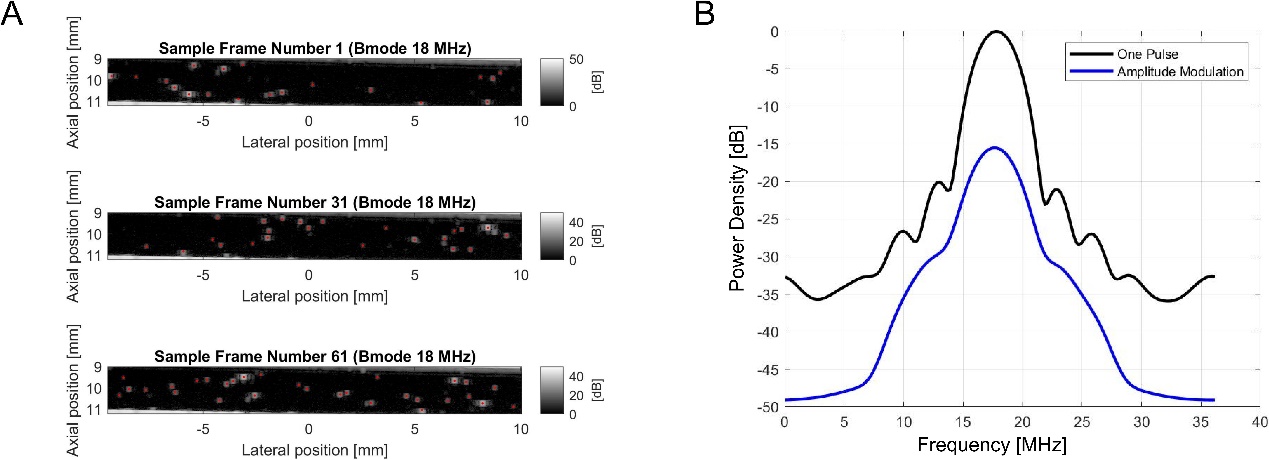


Figure S2 | Acoustic characterization of isolated PBCA NB. (A) Representative high-frequency (18 MHz) B-mode ultrasound frames of the flow phantom assay show spatial localization and tracking of individual NB flowing. (B) Averaged power density spectra computed from isolated NB signals. The black line ("One Pulse") represents the total baseline acoustic scattering (both linear and non-linear components). The blue line ("Amplitude Modulation") represents the isolated non-linear acoustic response extracted using an Amplitude Modulation (AM) sequence. The fundamental AM frequency spectrum shows incomplete signal cancellation, confirming that the rigid PBCA NB undergo asymmetric, non-linear oscillations under acoustic excitation. Higher harmonic frequencies cannot be captured due to the bandwidth limitations of the Vevo system.

**
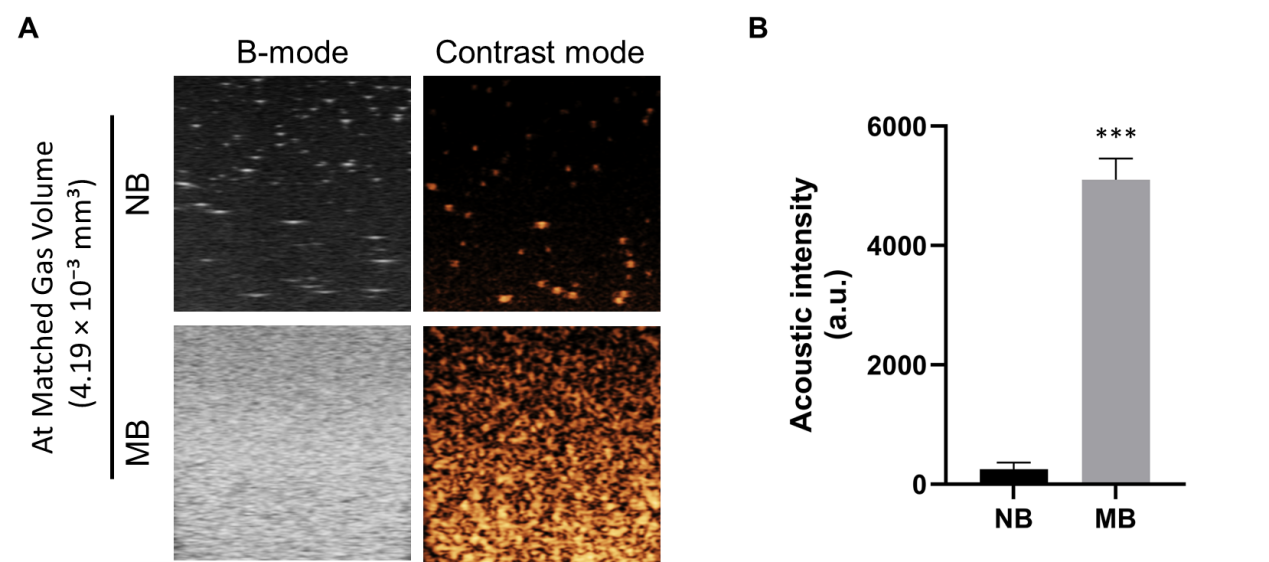
**

Figure S3 | Ultrasound echogenicity of PBCA NB and MB at matched gas volumes. (A) Representative B-mode and non-linear contrast mode US images (18 MHz preclinical setup) of PBCA NB and MB embedded in gelatin phantoms. To ensure a physically rigorous comparison, the concentrations of the suspensions were adjusted to contain an equivalent total estimated gas volume, derived from their respective size distributions. (B) Quantification of the acoustic intensity in contrast mode reveals that NB generate a lower overall acoustic signal compared to MB at a matched gas volume (*n* = 3). Data are presented as means ± SD. Statistical analysis was performed using unpaired two-tailed Student’s t-test. ****p* < 0.001.


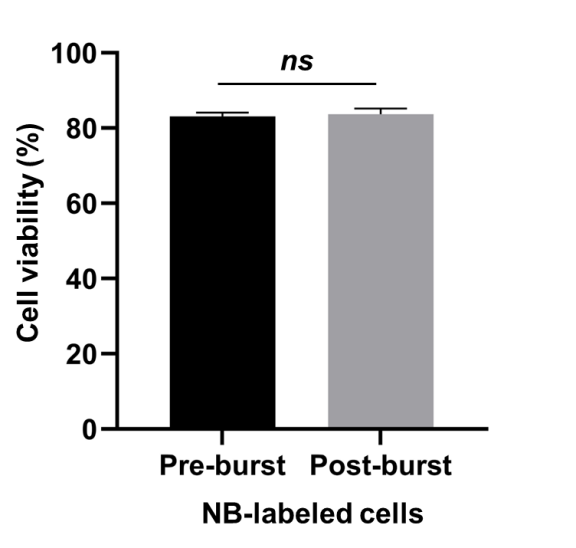


Figure S4 | Viability of NB-labeled macrophages after a destructive ultrasound pulse, measured by the XTT assay. A one-second-long destructive US pulse with, 100% intensity was applied (*n* = 3). No significant change in cell viability was observed, confirming that even high-pressure acoustic burst sequences do not compromise the biological integrity of NB-labeled cells. Data are presented as means ± SD. Statistical analysis was performed using unpaired two-tailed Student’s t-test. *ns = not significant*.


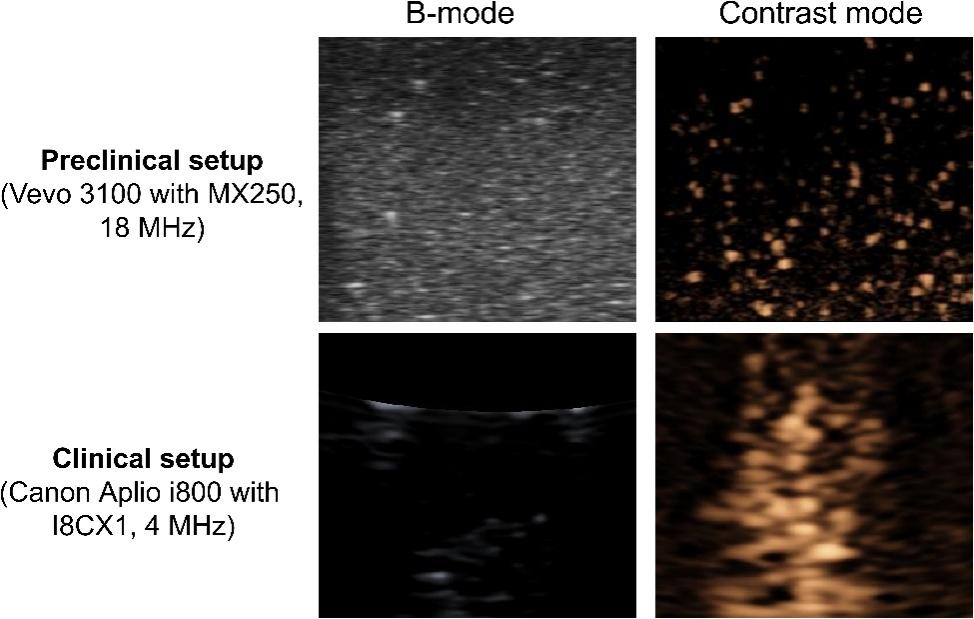


Figure S5 | In vitro US imaging of NB-labeled cells using preclinical and clinical systems. NB-labeled cells in a gelatine phantom (5*10^5^/mL) using the preclinical setup (Vevo 3100, 18 MHz), are highly echogenic in both fundamental B-mode and non-linear contrast mode. While their B-mode echogenicity is lower in the clinical setup (Canon Aplio i800, 4 MHz), the NB-labeled cells generate robust and clearly detectable signals in contrast mode. This indicates that intracellular PBCA NB provide sufficient acoustic signals in contrast mode at clinically relevant frequencies.


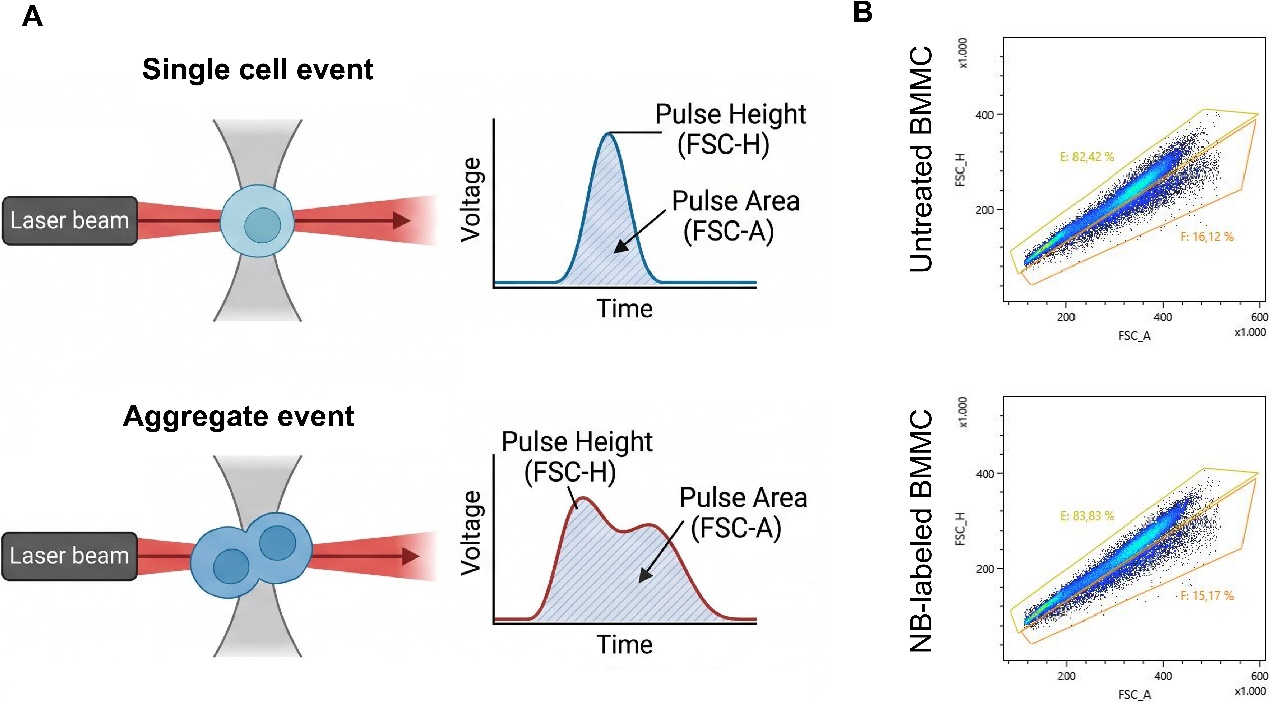


Figure S6 | Evaluation of cell aggregation following NB labeling using flow cytometry. (A) Schematic explaining the flow cytometry doublet discrimination principle. When a single cell passes through the interrogation laser, the resulting voltage pulse height (FSC-H) is proportional to its area (FSC-A). Conversely, when an aggregate or doublet passes through, the pulse area increases relative to the height. The figure was created with BioRender.com. (B) Representative histograms of flow cytometry scatter plots show that the populations within the diagonal gate (Gate E) represent single cells, while those in the lower right gate (Gate F) represent doublets or aggregates. NB-labeled cells exhibit a single-cell fraction (~83.8%) highly comparable to untreated control cells (>82%), quantitatively confirming that the intracellular NB labeling protocol does not induce cell aggregation.


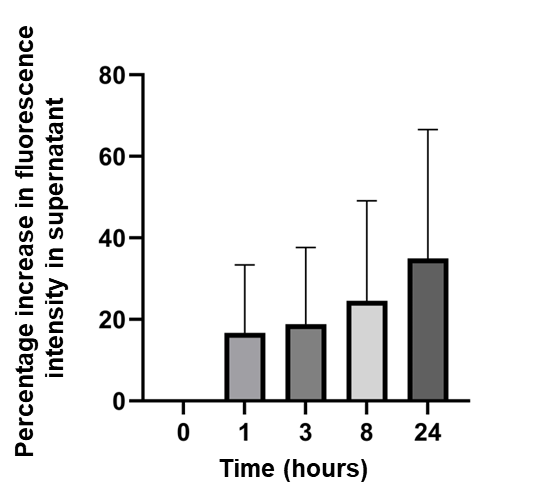


Figure S7 | Cellular excretion of internalized PBCA NB. The bar graph illustrates the percentage increase in fluorescence intensity (FI) detected in the cell culture supernatant at 1, 3, 8, and 24 hours post-labeling relative to baseline (0 h). The gradual, time-dependent accumulation of fluorescence in the extracellular space demonstrates that the cells actively process and excrete the internalized NB or their hydrolyzed polymeric fragments via exocytosis (*n* = 3). Data are presented as mean ± SD. The percentage increase in fluorescence intensity at each time point was calculated as following: FI_Increase_ (%) = [(FI_time point_ - FI_0h_) / FI_0h_ ] * 100%.

**
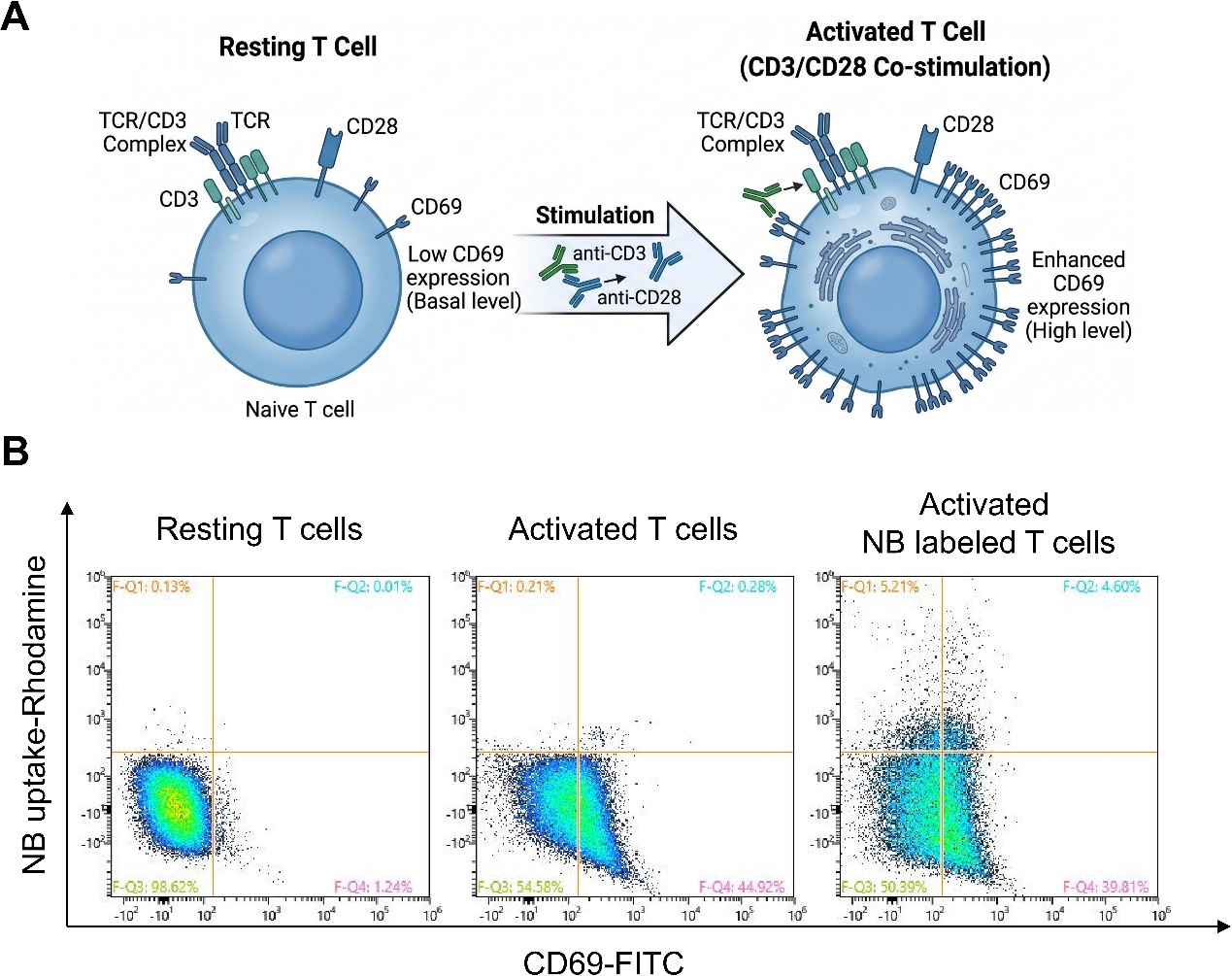
**

Figure S8 | Functional activation of T cells following intracellular NB labeling. (A) A schematic illustration of the in vitro T cell activation assay. Resting T cells exhibit low basal expression of the early activation marker CD69. Co-stimulation with anti-CD3 and anti-CD28 antibodies mimics physiological antigen recognition, triggering T cell receptor (TCR) signaling pathways and leading to enhanced surface expression of CD69. The figure was created with BioRender.com. (B) Flow cytometry analysis shows the activation capacity of unlabeled and NB-labeled T cells. Rhodamine-loaded NB were used to track cellular uptake (y-axis), while CD69-FITC was used to measure activation (x-axis). Unstimulated resting T cells show minimal CD69 expression (left panel). Upon CD3/CD28 stimulation, unlabeled T cells exhibit a robust upregulation of CD69 (middle panel). Following incubation with NB and subsequent stimulation, a distinct population of NB-labeled cells demonstrates strong CD69 expression, appearing as an enhanced double-positive population in Quadrant 2 (Q2, right panel). This confirms that cells successfully engulfing the NB retain their functional competence and activation pathways.


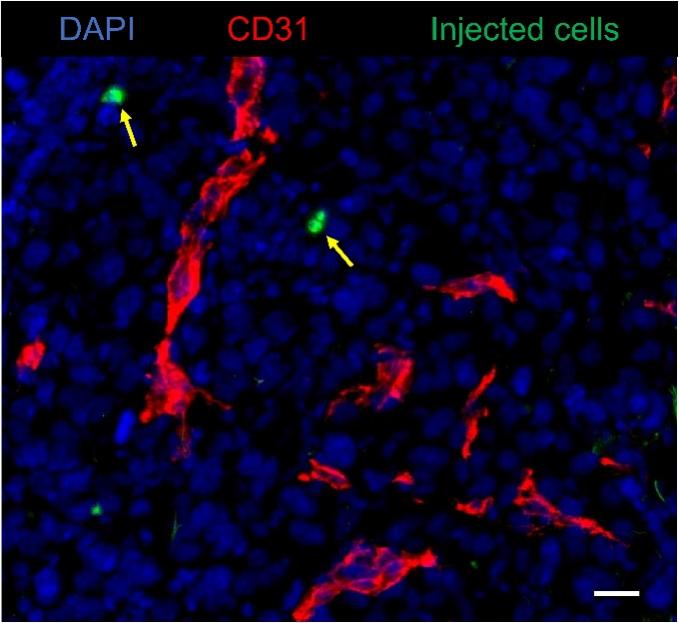


Figure S9 | Intravenously injected cells successfully extravasate into the tumor microenvironment. This representative immunofluorescence image shows tumor tissue harvested 6 hours after the intravenous injection of DiO-dyed and NB-labeled cells. Vessels are stained with CD31 (red) and cell nuclei are counterstained with DAPI (blue). Most DiO-dyed and NB-labeled cells (green, indicated by yellow arrows) are located outside of the vasculature, indicating their successful extravasation and infiltration into the tumor tissue. Scale bar = 20 µm.

Movie S1 | Nanoparticle tracking analysis (NTA) of PBCA NB. A representative NTA recording shows highly scattering, mobile NB with dynamic intensity fluctuations and diffuse light halos, which are characteristic of gas-filled nanostructures.

Movie S2 | ULM of 3D scanning sequence of NB-labeled cells under static conditions. A representative ULM processing sequence of NB-labeled cells under static conditions shows successfully localized echogenic events, which are indicated by red dots.

Movie S3 | Dynamic US imaging of NB labeled cells under flow. The representative US video acquired during perfusion of a flow phantom with NB-labeled cells shows moving echogenic events during real-time US acquisition.

Movie S4 | US imaging of the liver during intravenous injection of NB-labeled BMMC. The representative US video shows transition of labelled cells through the caval veins.

Movie S5 | US contrast-mode video of a subcutaneous tumor during infusion of unlabeled BMMC. The representative US video acquired during intra-arterial infusion of unlabeled BMMC into mice shows no detectable contrast signal within the tumor region.

Movie S6 | US contrast-mode video of a subcutaneous tumor during infusion of NB-labeled BMMC. The representative US video acquired during intra-arterial infusion of NB-labeled BMMC shows cell-related US signals moving within tumor vessels.
